# A systematic review and meta-analysis on antimicrobial resistance in marine bivalves

**DOI:** 10.3389/fmicb.2022.1040568

**Published:** 2022-12-01

**Authors:** Elisa Albini, Massimiliano Orso, Francesco Cozzolino, Luca Sacchini, Francesca Leoni, Chiara Francesca Magistrali

**Affiliations:** ^1^Istituto Zooprofilattico Sperimentale dell’Umbria e Delle Marche ‘Togo Rosati’, Perugia, Italy; ^2^Health Planning Service, Regional Health Authority of Umbria, Perugia, Italy

**Keywords:** antimicrobial resistance, antibiotic resistant bacteria, marine bivalves, food safety, MAR index

## Abstract

Bivalves are filter-feeding animals able to accumulate contaminants and microorganisms, either of marine or terrestrial origin. The aim of this study was to describe the prevalence of antimicrobial resistance (AMR) in bacterial isolates from bivalves using a systematic review of the literature. Comprehensive searches of MEDLINE, EMBASE, and Web of Science were carried out, based upon a registered protocol (PROSPERO), and following the preferred Reporting Items for Systematic reviews and Meta-Analysis (PRISMA) guidelines. The methodological quality of the included studies was assessed using a modified Hoy checklist. Meta-analyses of prevalence were carried out using random-effects models. In total, 103 articles were selected from 1,280 records and were included in the final analysis. The studies were from Asia (*n* = 54), Europe (*n* = 27), South and North America (*n* = 10 and *n* = 6, respectively), Africa (*n* = 2), Oceania (*n* = 1), and multicentre and intercontinental (*n* = 3). The meta-analysis of multiple antibiotic resistance (MAR) index revealed *Aeromonas* spp. as the genus with the highest prevalence of AMR (37%), followed by *Vibrio* spp. (34%), *Salmonella* spp. (18%), and *Escherichia coli* (15%). Resistance to third/fourth/fifth generation cephalosporins and fluoroquinolones, two highest priority, critically important antimicrobials (HPCIA), was recorded in approximately 10% of *E*. *coli* isolates. Resistance to carbapenems was very low (<2%) in *Salmonella* spp. and in *E*. *coli*, but was found in 5% of *Vibrio* spp. and in more than a third of *Aeromonas* spp. isolates. In aquatic bacteria, resistance to carbapenems was higher in Asian than in European isolates. Our study shows the presence of antibiotic resistant bacteria (ARB), including bacteria resistant to HPCIA, in marine bivalves, posing a risk for consumers.

## Introduction

Antimicrobial resistance (AMR) is the inability or reduced ability of an antimicrobial agent to inhibit the growth of a bacterium; in the case of a pathogenic microorganism, this can lead to therapeutic failure (EFSA, 2022). AMR is one of the 10 global public health threats facing humanity (WHO, 2021), estimated to be responsible for hundreds of thousands of deaths annually worldwide (O’Neill, 2016), with about 33,000 of these being in the European Union (EU) alone (Cassini et al., 2019). Humans can acquire antibiotic resistant bacteria (ARB) from many sources and routes, including *via* human (e.g., in hospitals or *via* the community settings), animal and environmental reservoirs (Holmes et al., 2016; Cassini et al., 2019). Antimicrobial resistant bacteria occurring in food-producing animals can spread to humans *via* the food-borne route (EFSA, 2022).

Antibiotic residues can reach aquatic environments through different sources, such as use in aquaculture (Schar et al., 2021), improper disposal of expired or unused medicines, and *via* agricultural run-off (Sanseverino et al., 2018). In the latter case, after their administration to livestock, antibiotics can end up in manure used as fertiliser and enter the aquatic system *via* surface runoff or by leaching to groundwater. Other possible routes include accidental spills and discharges during antibiotic manufacture or disposal on expiration. Furthermore, antibiotic residues have been found in treated effluents of wastewater treatment plants and/or receiving water bodies (Fonseca et al., 2020; Marano et al., 2020; Serra-Compte et al., 2021). Although the selective pressure of antibiotics disappears over time, resistance genes may be maintained by other selective forces. Indeed, other substances such as metals and biocides, if present in the environment, may favour resistance gene acquisition by co-selection (Baker-Austin et al., 2006; Wales and Davies, 2015), since determinants of resistance to such compounds are frequently harboured in the same mobile genetic elements as antibiotic resistance genes (Pal et al., 2015). Human- and terrestrial animal-derived gut bacteria, including those resistant to antimicrobials, can reach aquatic environments through different pathways (sewage systems, runoff from land, faeces from wild animals and birds, etc.), with the potential to contaminate seafood. Moreover, additional environmental selective pressure on the indigenous aquatic microflora, can be exercised by antibiotic residues, thus promoting horizontal gene transfer, maintenance or increase in antimicrobial resistance amongst the resident microbial population. Bivalve molluscan shellfish (e.g., clams, oysters, and mussels) are invertebrate animals that represent an important food commodity. They feed by filtering microalgae from the surrounding waters. However, during this activity they can also accumulate contaminants and microorganisms, such as naturally occurring or faecal derived bacteria (Lee and Silk, 2013), including those resistant to antimicrobial drugs. Frequently consumed raw or lightly cooked, bivalve molluscan shellfish can pose a risk for consumers.

Several studies have reported different antibiotic resistance patterns in isolates of different bacterial species (pathogenic or non-pathogenic to humans, autochthonous/allochthonous of the marine environment) recovered from bivalves throughout the world (Vignaroli et al., 2016; Grevskott et al., 2017; Mani et al., 2018; Lozano-Leon et al., 2019; Miotto et al., 2019; Parthasarathy et al., 2021). Nevertheless, a comprehensive systematic review on the status of AMR to the main antibiotic classes in bacterial isolates cultured from bivalves is not available.

The scope of this systematic review is to assess the prevalence of antibiotic resistance of bacterial isolates cultured from bivalves recovered from the environment, primary production or retail, and to evaluate the quality of the included studies to inform confidence in the reported results.

To this aim, available data on the presence of antibiotic-resistance in bacteria isolated from bivalve molluscs were critically summarised. This review includes studies on different bivalve species, collected in the environment, at the primary production or retail, from various geographical areas, and meta-analysis of AMR of isolates of bacterial indicators of faecal contamination (*E*. *coli*), zoonotic pathogens (*Salmonella*), as well as bacterial species naturally occurring in the marine environment (*Vibrio* spp., *Aeromonas* spp.). Data on methods used to evaluate AMR in different bacterial species from bivalves were also collected and analysed.

We used the systematic review and meta-analyses to identify knowledge gaps and provide information for the design of surveys and future surveillance on antimicrobial resistance in bacteria in bivalve molluscan shellfish.

## Materials and methods

### Scoping study and systematic review registration

The scope was to investigate the role of bivalves as carriers of antibiotic resistant bacteria, critically summarising available data on the presence of antibiotic-resistance in bacteria isolated from shellfish. The review included studies on different species of bivalves from various geographical areas and bacteria indicators of contamination from the terrestrial environment (e.g., *E*. *coli*) as well as bacterial species typical of the marine environment (e.g., *Vibrio* spp.).

This review is based on a study protocol registered in PROSPERO (registration number: CRD42020182165) and follows the Preferred Reporting Items for Systematic reviews and Meta-Analysis (PRISMA 2020) guideline (Page et al., 2021).

### Literature search strategy

Comprehensive searches of MEDLINE, EMBASE, and Web of Science were performed to identify published peer-reviewed articles, written in English or Italian, without publication date limitation. The search was conducted on the 14th April 2020, and an update was performed on 3rd September 2021. The search strategy was based on the Boolean combination of free text words and MeSH terms to identify records concerning (1) “bivalves,” (2) “antimicrobial resistance,” and (3) “bacterial species” (Appendix 1. Literature search strategies).

### Selection process and inclusion/exclusion criteria

The references screening was conducted in two steps. In the first step, two independent reviewers (EA, LS) conducted a title- and abstract-based screening to exclude researches irrelevant to the scope of the review, and any discrepancies were solved by discussion and consulting other review authors (CFM, FL). We included studies according to the following PICOS elements:

P (participants/population): studies on the prevalence of antimicrobial resistance in bacterial isolates from bivalves.
I (intervention/exposure): resistance to an antibiotic and/or an antibiotic class.
C (comparator): not applicable.
O (outcomes): (1): prevalence of resistance to the main antibiotic classes in selected bacterial species isolated from bivalves; (2): data on the methods used to detect antibiotic resistance in bacteria isolated from bivalves, including the bacterial species for which the assessment is made, the test used and its compliance to the internationally recognised methods for antibiotic susceptibility testing.
S (study design): cross-sectional studies and case reports.

We excluded studies on the antimicrobial resistance in other seafood, including crustaceans (as shrimps, crabs, and lobsters), fish (e.g., cod, salmon, and trout) or squid, turtles, octopus, and cuttlefish; all cooked seafood preparations were also excluded. Studies investigating the presence of antimicrobial resistance genes directly from the sample fall outside the scope of this review. Studies on prevalence of contaminants, including antibiotics have also been excluded. Regarding the study design, we excluded experimental or intervention studies, reviews, editorials, commentaries, notes, and conference proceedings. The reasons for exclusion were recorded for each reference screened (Appendix 2. Excluded studies).

### Quality assessment and the risk of bias

The methodological quality of the included studies was assessed by a modified version of the checklist developed by Hoy et al. (2012) or assessing risk of bias in prevalence studies. The modified checklist consists of eight items, two of which (item #4 and item #5) contain three sub-items. Each study received an overall risk of bias score from 0 to 8 points (the items #4 and #5 were considered fulfilled with at least two out of the three sub-items being evaluated as “yes”) and, according to this score, was judged as having a low (7–8 points), moderate (5–6 points) or high risk of bias (≤4 points). In addition, for each item of the checklist, we reported the percentages of “yes” and “no” answers.

Two review authors (EA, LS) independently assessed the study’s methodological quality, and any disagreement was resolved by other authors (FC, MO).

### Data extraction

Two independent reviewers (EA, LS) extracted data using standardised data collection forms previously tested on a sample of eligible articles and any disagreement was resolved by consensus and where necessary, by consulting other review authors (CFM, FL).

Information collected was related to prevalence of each resistant bacteria species (e.g., the number of samples/isolates collected/tested divided by the number of positive samples/isolates tested for), culture and identification methods, phenotypic AMR, laboratory testing methodologies used and the interpretation criteria, and the area of origin of the sample either retail, environmental, or production. For the phenotypic AMR, isolates where categorised as susceptible/resistant (non-susceptible), with those having intermediate and/or resistant results in the original papers being considered here as resistant (non-susceptible). The prevalence of AMR to a class was estimated for each study and bacterial genus or species, by dividing the number of resistant isolates to at least one antibiotic of the class by the total number of isolates tested, as already described (Jans et al., 2018). In addition, bibliographical details (e.g., author, year, language, and funding sources) were recorded. All data were organised into an Excel document (Microsoft Office, Microsoft, Redmond, WA, United States). The antimicrobials included in the data collection, classified following the WHO AWaRe classification of antibiotics (World health organization, 2018), are listed in Appendix 3.

### Statistical analysis

The data are expressed as a pooled estimate of prevalence of resistance for each antibiotic class. Data analysis was performed using STATA/SE 13. Meta-analyses of prevalence were carried out using random-effects models. Summary estimates were provided along with 95% confidence intervals (95% CIs). Heterogeneity amongst studies was assessed using I^2^ statistics. Possible sources of heterogeneity between studies were investigated through subgroup analyses by continent of origin of the samples. A value of *p* < 0.05 was considered significant in all of the analyses.

The multiple antibiotic resistance (MAR) index was calculated for *Salmonella* spp., *E*. *coli*, *Aeromonas* spp., *Vibrio* spp., *V*. *parahaemolyticus*, and *V*. *cholerae*, as the ratio between the number of resistant bacterial isolates and the total number of combinations tested (number of antibiotics classes * number of isolates tested) for each bacterial genus or species. MAR indices can be used as indicators of the risk of AMR increase.

Studies were included in the meta-analyses if they met the following criteria: at least 30 total isolates for all studies included in each meta-analysis; at least five isolates for each study; at least three studies for each meta-analysis or for each subgroup.

## Results

### Search results and descriptive analysis of the included studies

**Figure 1:**
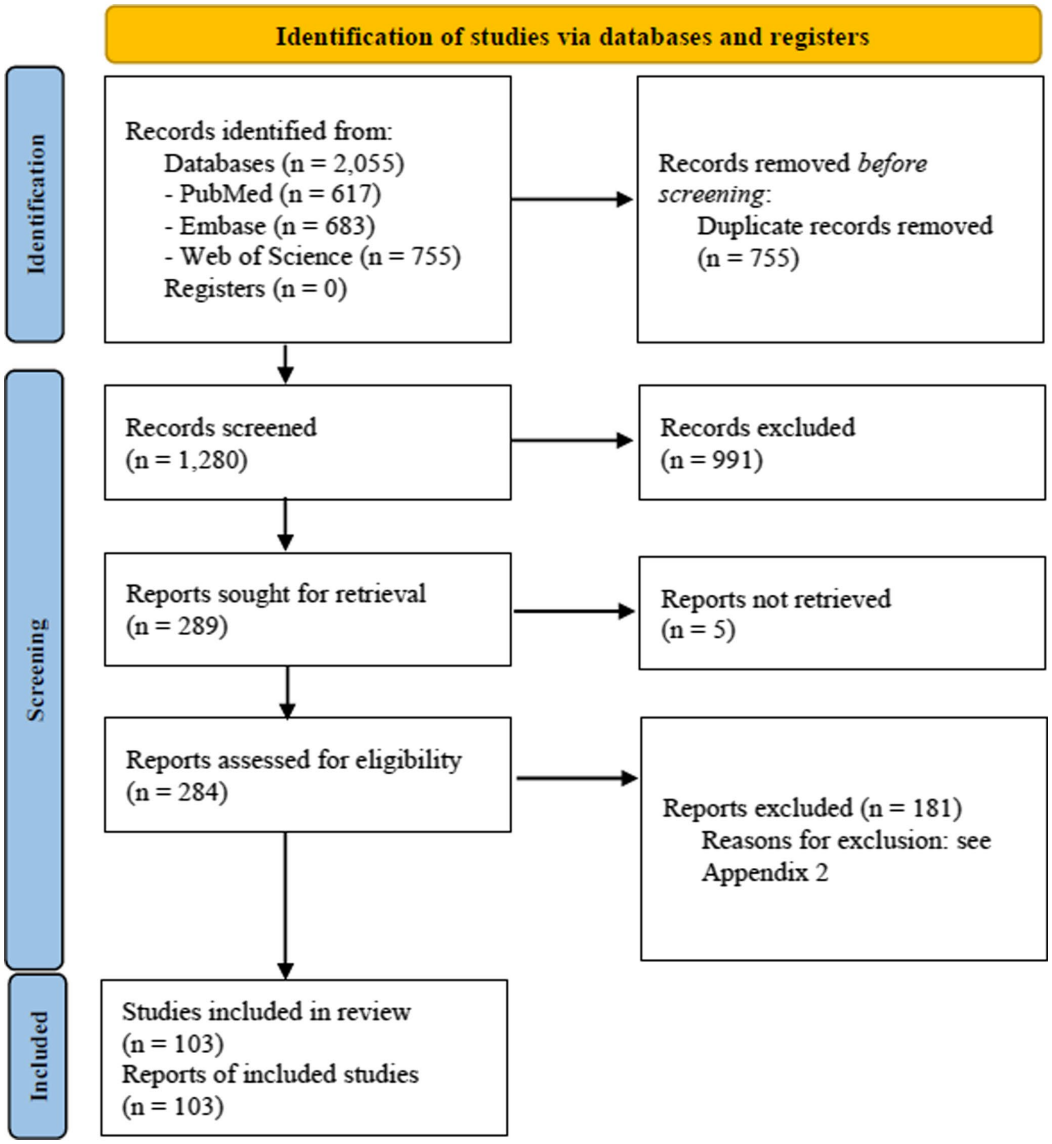
PRISMA 2020 flow diagram.

The literature search process is summarised in Figure 1 (PRISMA 2020 flow diagram).

In total, 2,055 references were retrieved from the electronic databases. After duplicates removal, the 1,280 remaining records were evaluated for titles and abstract. Following the title/abstract screening, 284 articles were assessed in full-text, of these 103 articles were included in the final analysis (Figure 1, PRISMA 2020 Flow Diagram). A list of the excluded articles and the reasons for exclusion are reported in Appendix 2.

The included studies (Appendix 4. Characteristics of included studies) collected and tested samples in 40 countries, representing all continents. Asia was the most frequent sampling location (*n* = 54, 52%), followed by Europe (*n* = 27, 26%) and South and North America (*n* = 10, 10% and *n* = 6, 6%, respectively). Only 2 (2%) studies were located in Africa, 1 study (1%) was performed in Oceania, whilst three studies were multicentre and intercontinental. Specifically, the most frequent sampling locations were Korea (*n* = 19 studies), China (*n* = 9), Brazil (*n* = 8), Italy (*n* = 7), India, Spain, and Malaysia (*n* = 6 each), United States (*n* = 4 each), Thailand (*n* = 4), France, Germany, and Vietnam (*n* = 3 each), Kuwait, Norway, Canada, Taiwan, and Croatia (*n* = 2 each), and Tunisia (*n* = 1). The less frequent sampling locations were Australia, Chile, Denmark, Ecuador, Greece, Iran, Japan, Mozambique, Qatar, and Northern Ireland with only one study each.

Regarding the distribution of bacterial genera isolated from bivalves, the studies comprised in the systematic review investigated AMR of 22 bacterial genera, including both Gram-positive and Gram-negative bacteria. The main bacterial genus investigated was *Vibrio* spp., which was described in 45 studies, which analysed bivalves produced or collected in Africa, Asia, Europe, North, and South America. Others frequently investigated bacterial genera were *Salmonella* (13 studies with samples produced or harvested in Africa, Asia, Europe, and North America), *E*. *coli* (12 studies from all continents, except Africa), *Aeromonas* spp. (10 studies which sampled bivalves in Asia, Europe, and South America), and *Enterococcus* spp. (five studies performed in Europe and South America).

Most of the studies investigated AMR in samples purchased at local markets or supermarkets (*n* = 41), whilst in 30 studies samples were harvested from the environment. Furthermore, 13 studies investigated AMR in samples from aquaculture, whilst 16 studies included samples of mixed origins: aquaculture/environment (*n* = 7); environment/retail (*n* = 5); retail/aquaculture (*n* = 3); and retail/aquaculture/environment (*n* = 1). Lastly, three studies did not specify the origin of the samples.

### Prevalence of AMR in bacteria

The meta-analyses were performed only for *Salmonella* spp., *E*. *coli*, *Aeromonas* spp., and *Vibrio* spp. Given the high number of studies investigating AMR in the latter genus, meta-analyses were also carried out for the species *V*. *parahaemolyticus* and *V*. *cholerae*.

Tables 1A,B show the number of studies included in the meta-analysis, the proportion of resistant isolates, 95% Confidence Interval (CI) and I^2^ values for each antibiotic class.

**Table 1A:** Number of studies included in the meta-analysis, proportion of resistant isolates (%), 95% Confidence Interval (CI) and I^2^ values for each antibiotic class.

**Antibiotic class**	***Vibrio* spp*.***				***V. parahaemolyticus***				***V. cholerae***			
	**N. studies**	**%**	**CI95%**	**I** ^**2**^ **(%)**	**N. studies**	**%**	**CI95%**	**I** ^**2**^ **(%)**	**N. studies**	**%**	**CI95%**	**I** ^**2**^ **(%)**
Amphenicols	36	0	0-1	73	21	0	0-1	54	3	0	0-1	0
Aminoglycosides	41	34	21-47	98	23	35	14-58	99	3	13	0-40	93
Carbapenemes	16	5	1-11	91	7	0	0-0	0	-	-	-	-
Carboxypenicillins	4	92	64-100	93	-	-	-	-	-	-	-	-
Quinolones	26	3	1-6	86	14	3	0-6	81	-	-	-	-
Fluoroquinolones	36	9	5-14	94	22	10	4-17	94	-	-	-	-
First/Second generation cephalosporins	26	62	41-80	99	14	69	49-87	97	-	-	-	-
Third/Fourth/Fifth generation cephalosporins	30	16	8-27	97	17	22	8-41	97	-	-	-	-
Beta lactam - beta lactamase inhibitor	7	6	0-21	95	3	6	0-30	97	-	-	-	-
Lincosamides	5	83	66-95	78	-	-	-	-	-	-	-	-
Glycopeptides	14	77	52-95	97	9	84	52-100	97	-	-	-	-
Macrolides	21	57	37-76	99	9	75	50-94	97	-	-	-	-
Nitrofurans	3	33	0-81	90	-	-	-	-	-	-	-	-
Penicillins	41	83	72-92	98	23	91	77-99	98	3	7	0-26	90
Polypeptides	3	72	40-95	91	-	-	-	-	-	-	-	-
Rifamycins	9	84	77-91	65	4	88	76-96	79	-	-	-	-
Sulphonamides	7	25	5-52	99	-	-	-	-	-	-	-	-
Trimethoprim - sulfonamide combinations	28	3	1-6	87	14	5	1-11	87	-	-	-	-
Tetracyclines	37	12	6-20	97	21	7	3-14	93	3	6	0-36	95
Trimethoprim	6	21	4-44	97	3	34	10-64	98	-	-	-	-
Multiple antibiotic resistance (MAR)	41	32	25-39	99	23	34	28-41	99	3	9	5-15	92

**Table 1B:** Number of studies included in the meta-analysis, proportion of resistant isolates (%), 95% Confidence Interval (CI) and I^2^ values for each antibiotic class.

**Antibiotic class**	***Escherichia coli***				***Salmonella* spp*.***				***Aeromonas* spp*.***			
	**N. studies**	**%**	**CI95%**	**I** ^**2**^ **(%)**	**N. studies**	**%**	**CI95%**	**I** ^**2**^ **(%)**	**N. studies**	**%**	**CI95%**	**I** ^**2**^ **(%)**
Amphenicols	7	10	1-24	93	8	8	2-18	78	7	2	0-8	67
Aminoglycosides	9	11	4-22	93	10	11	1-27	93	7	15	2-36	90
Carbapenemes	4	0	0-1	0	3	0	0-3	0	7	36	5-77	98
Carboxypenicillins	-	-	-	-	-	-	-	-	-	-	-	-
Quinolones	6	7	1-17	91	10	22	4-45	94	4	41	0-92	97
Fluoroquinolones	7	11	2-25	95	9	2	0-10	80	7	1	0-8	80
First/Second generation cephalosporins	7	22	0-61	99	3	60	6-100	93	6	75	36-99	97
Third/Fourth/Fifth generation cephalosporins	5	10	3-21	91	7	2	0-7	25	8	13	4-23	85
Beta lactam - beta lactamase inhibitor	3	20	2-49	97	8	16	3-34	84	-	-	-	-
Lincosamides	-	-	-	-	-	-	-	-	-	-	-	-
Glycopeptides	-	-	-	-	-	-	-	-	-	-	-	-
Macrolides	-	-	-	-	-	-	-	-	4	41	0-92	97
Nitrofurans	3	22	0-80	99	4	35	0-95	97	-	-	-	-
Penicillins	8	34	13-59	98	10	24	4-53	97	8	100	100-100	0
Polypeptides	-	-	-	-	-	-	-	-	-	-	-	-
Rifamycins	-	-	-	-	-	-	-	-	4	89	79-96	51
Sulphonamides	4	20	0-56	94	-	-	-	-	-	-	-	-
Trimethoprim - sulfonamide combinations	7	16	5-30	95	11	13	1-33	95	7	18	2-44	95
Tetracyclines	8	12	5-22	86	9	47	19-76	97	8	19	2-43	94
Trimethoprim	3	6	2-11	0	-	-	-	-	-	-	-	-
Multiple antibiotic resistance (MAR)	9	15	8-25	99	11	18	11-26	97	9	37	25-50	98

Typical of the marine environment, *Vibrio* was the main bacterial genus investigated. Resistance frequencies were diverse between antimicrobial classes and these were very high for carboxypenicillins (92%,), glycopeptides (77%), first/second generation cephalosporins (62%), lincosamides (83%), penicillins (83%), polypeptides (72%) rifamycins (84%), and macrolides (57%). High levels of AMR were observed for aminoglycosides (34%), nitrofurans (33%), sulphonamides (25%), and trimethoprim (21%). The levels of resistance to third/fourth/fifth generation cephalosporins (16%) and tetracyclines (12%) were moderate. None or low AMR was observed for amphenicols, carbapenems, quinolones, fluoroquinolones, beta lactam − beta lactamase inhibitors, and trimethoprim − sulfonamide combinations.

The prevalence of AMR in *V*. *parahaemolyticus* overlapped that of *Vibrio* spp., except for the carboxypenicillins, lincosamides, nitrofurans, polypeptide, and sulphonamides, which were not included in the analysis. *Vibrio cholerae* was described only in three studies, with a low or very low prevalence of AMR for amphenicols, penicillins, and tetracyclines. In *V*. *cholerae*, the highest AMR prevalence was recorded for aminoglycosides (13%).

*Escherichia coli* was the second most described bacterial species. Overall, AMR prevalences were low for all the antimicrobial classes analysed. The highest prevalence was observed for penicillins (34%), whilst total susceptibility was observed for carbapenems.

The meta-analyses of *Salmonella* showed a low prevalence of resistance for all antibiotic classes, although it was serovar specific. A very high prevalence was observed for first/second generation cephalosporins (60%). Prevalences of resistance were high for tetracyclines (47%), nitrofurans (35%), and quinolones (22%). An almost complete susceptibility was observed for fluoroquinolones (2%) and third/fourth/fifth generation cephalosporins (2%).

The results of the AMR prevalence meta-analysis performed on *Aeromonas* spp. were diverse and very high prevalences were observed for rifamycins (89%) and first/second generation cephalosporins (75%), and a complete resistance to penicillins. Very low AMR was observed for amphenicols (2%) and fluoroquinolones (1%).

The meta-analyses of MAR highlighted that *Aeromonas* spp. is the genus with the highest prevalence of multiple antibiotic resistance bacteria (37%), followed by *Vibrio* spp. (32%). However, the MAR of the latter bacterial genus depends on the bacterial species: in fact, *V*. *parahaemolyticus* has a higher prevalence of MAR than *V*. *cholerae* (34% and 9%, respectively). The meta-analyses of MAR of *E*. *coli* and *Salmonella* spp. are similar (15% and 18%, respectively).

The vast majority of meta-analyses showed high heterogeneity (Tables 1A,B), except few meta-analyses in which the prevalence of AMR was close to zero (*V*. *parahaemolyticus vs* carbapenems; *V*. *cholerae vs* amphenicols; *E*. *coli vs* carbapenems and trimethoprim; *Salmonella* spp. *vs* carbapenems and third/fourth/fifth generation cephalosporins), or close to 100% (*Aeromonas* spp. *vs* penicillins).

We performed subgroup meta-analyses to investigate possible geographical differences in AMR resistance in *Vibrio* spp., *V*. *parahaemolyticus*, *Aeromonas* spp., and *Salmonella* spp. and the results are shown in Tables 2A,B.

**Table 2A:** Number of studied included in the meta-analysis, proportion of resistant isolates (%), 95% Confidence Interval (CI) and I^2^ values for each antibiotic class.

	*Vibrio* spp.												*V. parahaemolyticus*							
	Europe				Asia				South America				Asia				South America			
Antibiotic class	N. studies	%	CI 95%	I^2^(%)	N. studies	%	CI 95%	I^2^(%)	N. studies	%	CI 95%	I^2^(%)	N. studies	%	CI 95%	I^2^(%)	N. studies	%	CI 95%	I^2^(%)
Amphenicols	5	0	0-0	0	25	1	0-3	76	3	5	0-25	-	14	0	0-1	52	3	5	0-25	-
Aminoglycosides	6	50	15-84	97	29	39	21-59	98	3	4	1-9	-	16	45	17-75	99	3	4	1-9	-
Carbapenemes	3	0	0-1	-	10	11	2-24	94	-	-	-	-	-	-	-	-	-	-	-	-
Quinolones	-	-	-	-	19	6	2-11	86	3	2	0-6	-	10	4	1-10	85	3	2	0-6	-
Fluoroquinolones	6	0	0-2	0	24	17	9-26	95	3	4	1-9	-	15	16	6-30	96	3	4	1-9	-
Third/Fourth/Fifth generation cephalosporins	4	1	0-8	59	22	23	10-39	97	-	-	-	-	-	-	-	-	-	-	-	-
Beta lactam - beta lactamase inhibitor	3	0	0-1	-	4	14	0-42	97	-	-	-	-	-	-	-	-	-	-	-	-
Penicillins	6	53	17-86	97	29	87	71-97	98	3	98	93-100	-	16	90	69-100	99	3	98	93-100	-
Trimethoprim - sulfonamide combinations	4	0	0-1	0	20	5	2-10	86	-	-	-	-	-	-	-	-	-	-	-	-
Tetracyclines	5	0	0-0	13	26	21	10-35	97	3	4	1-9	-	14	14	5-26	95	3	4	1-9	-

**Table 2B:** Number of studied included in the meta-analysis, proportion of resistant isolates (%), 95% Confidence Interval (CI) and I^2^ values for each antibiotic class.

	***Salmonella spp.***									***Aeromonas spp.***								
	**Europe**					**Asia**				**Europe**					**Asia**			
**Antibiotic class**	**N. studies**	**%**	**CI** **95%**	**I** ^**2**^ **(%)**		**N. studies**	**%**	**CI** **95%**	**I** ^**2**^ **(%)**	**N. studies**	**%**	**CI** **95%**	**I** ^**2**^ **(%)**		**N. studies**	**%**	**CI** **95%**	**I** ^**2**^ **(%)**
Aminoglycosides	3	31	0-88	-		4	13	0-44	89	-	-	-	-		-	-	-	-
Carbapenemes	-	-	-	-		-	-	-	-	3	4	0-28	-		4	67	33-94	94
Quinolones	3	8	2-15	-		4	47	1-97	96	-	-	-	-		-	-	-	-
Third/Fourth/Fifth generation cephalosporins	3	8	1-17	-		3	0	0-3	-	3	26	3-61	-		4	7	1-17	69
Penicillins	3	22	11-36	-		4	9	0-35	85	3	100	100-100	-		5	100	99-100	0
Trimethoprim - sulfonamide combinations	3	12	5-20	-		4	38	0-95	97	-	-	-	-		-	-	-	-

We observed a higher prevalence of resistance to tetracyclines (21%, 0%, and 4%, respectively) in *Vibrio* spp. isolated from Asia than from Europe and South America. The *Vibrio* spp. isolated from Asia showed higher prevalence to carbapenems (11% and 0%, respectively), fluoroquinolones (17% and 0%, respectively), third/fourth/fifth generation cephalosporins (23% and 1%, respectively), and trimethoprim − sulfonamide combinations (5% and 0%, respectively) comparing to European isolates.

The bacteria isolated from South America presented lower prevalence of resistance to aminoglycosides compared to the bacteria isolated from Europe and Asia (4%, 50%, and 39%, respectively).

Geographical differences in AMR resistance investigated in *V*. *parahaemolyticus* highlighted a higher prevalence to aminoglycosides in isolates from Asia than from South America (45% *vs*. 4%, respectively), whilst no differences were observed for amphenicols, fluoroquinolones, quinolones, penicillins, and tetracyclines.

*Salmonella* spp. isolated from Asian countries had the same prevalence of resistance as European isolates.

No difference in prevalence of AMR was observed in *Aeromonas* spp. isolated from Asia or from Europe, except for carbapenems (67% and 4%, respectively).

### Quality assessment and the risk of bias

The methodological quality of studies for AMR in bivalves was assessed by using the checklist of Table 3.

**Table 3:** Quality assessment.

	**Answers**	
**Item**	Total number of low risk answers/Total number of answers (%)	Total number of high risk answers/ Total number of answers (%)
1. Was the study’s sample a close representation of the species of bivalves of a certain geographical area?	1/103 (1%)	102/103 (99%)
2. Was some form of random selection used to select the sample?	10/103 (10%)	93/103 (90%)
3. Was the sample size adequate?	2/103 (2%)	101/103 (98%)
4. Was an acceptable case definition used in the study?		
a. Is it possible to trace the AMR results back to the species of bivalves of origin?	69/103 (67%)	34/103 (33%)
b. Is the site of sample collection (retail vs production sites) fully described?	100/103 (97%)	3/103 (3%)
c. Is the period of sample collection identified?	81/103 (79%)	22/103 (21%)
5. Was the study instrument that measured the parameter of interest shown to have reliability and validity (if necessary)?		
a. Did the authors refer to CLSI or EUCAST guidelines for antimicrobial susceptibility testing?	85/103 (83%)	18/103 (18%)
b. Did the authors use a control strain for antimicrobial susceptibility testing?	46/103 (45%)	57/103 (55%)
c. Was the source of the breakpoints for interpretation clearly described and was this source a CLSI or an EUCAST document?	61/103 (59%)	42/103 (41%)
6. Was the same mode of data collection used for all subjects?	100/103 (97%)	3/103 (3%)
7. Was the length of the shortest prevalence period for the parameter of interest appropriate?	33/103 (32%)	70/103 (68%)
8. Were the numerator(s) and denominator(s) for the parameter of interest appropriate?	102/103 (99%)	1/103 (1%)

In the majority of the studies the sample collection (retail *vs*. production site) was fully described (97%, 100 out of 103) and it was possible to trace back AMR results to the species of bivalve of origin (67%, 69 out 103). Furthermore, 85 (83%) of the 103 studies used CLSI or EUCAST guidelines for antimicrobial susceptibility testing, 61 (59%) used clearly described breakpoints for interpretation of AMR and 46 (45%), used a control strain for AST. As for the items 4 and 5, the number of studies satisfying at least 2/3 sub items was 92% and 66%, respectively. The length of the period of sampling was at least 1 year in 32% of the studies (Table 3). The total number of tested and resistant isolates was clearly reported in 99% (102 out of 103) of the studies (Item 8 of Table 3).

Methodological biases were identified particularly in the representativeness of the sample (Items 1–3 of Table 3). The overall bias score was of high, moderate and low quality in 1% (1 out of 103), 28% (29 out of 103), and 71% (73 out of 103) of studies, respectively.

## Discussion

In this study, we report the prevalence of antibiotic resistance in marine bivalves through the systematic analysis of point prevalence studies. We collected and analysed data on the prevalence of antibiotic resistance in bacteria originating from the terrestrial environment, such as *E*. *coli*, as well as zoonotic bacteria such as *Salmonella* spp., and also data regarding aquatic bacteria, *Aeromonas* spp. and *Vibrio* spp. Bivalves filter large volumes of seawater and can retain up to 90% of the suspended particles, including bacteria of different origins, from the water column (Beyer et al., 2017; Baralla et al., 2021). Whilst *Aeromonas* spp. and *Vibrio* spp. are autochthonous of aquatic environments, Enterobacteriaceae originate from humans or terrestrial animals through runoff or sewage or costal wildlife (Grevskott et al., 2017; Vignaroli et al., 2018). Thus, bivalves are ideal indicators for faecal contamination of coastal environments (Vignaroli et al., 2016; Grevskott et al., 2017). The monitoring of antibiotic-resistant terrestrial bacteria in bivalves could give important indications regarding the circulation of resistant bacteria in the population residing in the basin of the area of origin.

In our study, high rates of resistance in *E*. *coli* were observed for sulphonamides, nitrofurans, and beta-lactams, including penicillins, first/second generation cephalosporins, and beta lactam-beta lactamase inhibitors. We found moderate levels of resistance to trimethoprim – sulphonamides combination, fluoroquinolones, aminoglycosides, and tetracyclines. This is a common resistance profile in *E*. *coli* because of the long-term use of these molecules in human and veterinary medicine (Van Boeckel et al., 2019; EFSA, 2022). Resistance to third/fourth/fifth generation cephalosporins and fluoroquinolones, two antibiotic classes recognised as highest priority, critically important antimicrobials (HPCIA) in human medicine (WHO, 2018), was recorded in approximately 10% of the isolates. *E*. *coli*, with an approximate 800,000 attributable deaths in 2019, is considered the lead pathogen causing death associated with AMR in humans worldwide (Murray et al., 2022). The same authors estimated that fluoroquinolones-resistant and third generation-resistant *E*. *coli* caused between 50,000 and 100,000 resistance-attributable deaths in 2019 each. Our findings suggest that bivalves may be carriers of *E*. *coli* resistant to HPCIA, posing a possible health risk to consumers in the case of bivalve molluscs intended for human consumption, particularly when eaten raw. Indeed, *E*. *coli* isolates resistant to third/fourth/fifth generation cephalosporins were recovered from bivalves collected from the environment (Al-Sarawi et al., 2018; Miotto et al., 2019; Divya et al., 2020; Jeong et al., 2021), moreover from bivalve molluscan shellfish purchased at the market (Yu et al., 2020). In addition, ESBL producing *E*. *coli* or other Enterobacteriaceae were reported in different types of bivalves sampled in markets in Tunisia (Mani et al., 2018) and Germany (Vu et al., 2018). For *Salmonella* spp., we found a high prevalence of resistance to quinolones, first/second generation cephalosporins, tetracyclines, penicillins, and nitrofurans and moderate levels of resistance to aminoglycosides, beta lactams - beta lactamase inhibitors, and trimethoprim - sulfonamide combinations. By contrast, resistance to fluoroquinolones and third/fourth/fifth generation cephalosporins was low and resistance to carbapenems was very rare. It should be noted that antibiotic resistance in *Salmonella* spp. varies according to the serotype to which it belongs (EFSA, 2018). The prevalence of antibiotic resistance in this bacterial species in a population is strongly dependent on the dissemination of successful clones (EFSA, 2021).

All *Aeromonas* isolates were resistant to penicillin. This is in agreement with the findings reported by other authors, who have indicated a natural resistance to penicillins and first/second generation cephalosporins of this genus (Fernández-Bravo and Figueras, 2020; Lee et al., 2020). Unfortunately, we could not discriminate the prevalence of resistance in different species of the genus *Aeromonas*. This was due to the limited number of studies included in our investigation, which prevented the possibility to estimate the prevalence of antibiotic resistance at the species level. Moreover, it should be noted that species identification is cumbersome in *Aeromonas* spp. (Baron et al., 2017). Therefore, comparing data about antibiotic resistance at the genus rather than at the species level seemed to us to be more reliable for comparing isolates from different studies. Resistance to third/fourth/fifth generation cephalosporins was detected in 13% of *Aeromonas* isolates. Such resistance is inducible in *Aeromonas* spp. and linked to the presence of beta lactamase, as class B metallo-β-lactamase, class C cephalosporinase, and class D penicillinases (Fernández-Bravo and Figueras, 2020). In this genus, beta lactamases are often encoded by plasmids, posing a threat for the possible transfer to other bacterial species *via* horizontal gene transfer (Piotrowska et al., 2017). Resistance to carbapenems was found in more than a third of *Aeromonas* spp. isolates. Once again, such resistance is probably due to the presence of metallo-β-lactamase and particularly of *cph*A-related genes (Piotrowska et al., 2017). The high resistance rates found in isolates from bivalves are in accordance with what has already been reported by Schar (Schar et al., 2021) in isolates of fish origin in Asia. In our study, we found a higher prevalence of resistance to carbapenems in Asia than in Europe. Since carbapenems are not used in aquaculture, the high resistance rates to carbapenems may be the consequence of the use of beta-lactams in this sector (Schar et al., 2021). Our results confirm concerns about the role of *Aeromonas* spp. in the spread of β-lactamases into the natural environment. High levels of resistance were also found for quinolones, macrolides and rifamycins whilst moderate levels were found for tetracyclines, trimethoprim-sulphonamides, and aminoglycosides. Overall, *Aeromonas* spp. isolates showed the highest MAR index, confirming the importance of this genus as a reservoir of antibiotic-resistance genes (Gabashvili et al., 2022).

In *Vibrio* spp., a very high prevalence of AMR was found to beta-lactams (penicillin, carboxypenicillins, first/second generation cephalosporins), lincosamides, glycopeptides, macrolides, polypeptides, and rifamycins. High prevalence of AMR were recorded for aminoglycosides, sulfonamides and nitrofurans. Again, we observed a higher prevalence of AMR in isolates from Asia than from Europe and South America, apart for aminoglycosides, for which resistance was higher in isolates from Europe than from the other continents. Worryingly, resistance to carbapenems, which was recorded in 5% of isolates, was also higher in Asian than in European isolates. Of *Vibrio* spp., *V*. *parahaemolyticus* is a species that can be part of the natural biota of bivalve molluscs, but can also be a human pathogen responsible for gastroenteritis associated with consumption of raw or undercooked seafood, particularly shellfish. The majority of *V*. *parahaemolyticus* strains in the environment do not harbour the genes coding for the major virulence factors, the thermostable direct haemolysin (TDH) and the TDH-related haemolysin (TRH; DePaola et al., 2000; Robert-Pillot et al., 2004; Rodriguez-Castro et al., 2010; Ottaviani et al., 2013). The prevalence of AMR in *V*. *parahaemolyticus* isolates from bivalves was similar to that found for *Vibrio* spp. Independently from the continent of isolation, resistance to penicillins was high in *V*. *parahaemolyticus* isolates from bivalves. This finding is in agreement with a previous review (Elmahdi et al., 2016), that examined antibiotic resistance in *V*. *parahaemolyticus* isolates of different origin (clinical and environmental) and found that, regardless of the country of isolation, antibiotic resistance to penicillin, ampicillin, and tetracycline were most frequently observed in isolates of this bacterial species. Such resistance is probably linked to the presence of a class A carbenicillin-hydrolyzing β-lactamase (CARB; Chiou et al., 2015; Zhao et al., 2018). Some geographical differences were observed for tetracyclines, and fluoroquinolones, with a prevalence of resistance that was moderately higher in isolates from Asia compared to studies for other continents, and aminoglycoside, where, conversely, a higher percentage of resistance was found in isolates from Europe than from Asia. Thankfully, our study confirms the susceptibility of *Vibrio* spp. and *V*. *parahaemolyticus* to tetracyclines, amphenicols, and quinolones, three antibiotic classes recommended for the treatment of vibriosis (Silva et al., 2018).

Antibiotic resistance was generally higher in marine than in terrestrial bacteria, as outlined by the analysis of the MAR indices. A similar trend was observed by Liu (Liu et al., 2019) in shrimps, where the majority of antimicrobial resistance genes ARGs were found to originate from mobile elements carried by *Vibrio* spp. Recently, Gabashvili et al. (2022) showed that *Aeromonas* spp. and *Vibrio* spp. are amongst the most important reservoir of ARGs in the marine environment. Here, these two bacterial genera can transfer ARGs to other bacterial genera through horizontal gene transfer. Since bivalves concentrate different species of bacteria together with antibiotic residues, they provide an ideal setting for the exchange of resistant determinants (Grevskott et al., 2017) and a possible vector for the transmission of emerging antibiotic determinants from the environment to humans (Lozano-Leon et al., 2019).

Regardless of the bacterial genus, we found higher resistance rates in Asian than in European isolates. Reverter et al. (2020) reported high resistance levels in fish pathogens in economically vulnerable regions, including Southeast Asia. Other authors reported the same outcome in sewage samples and found a correlation between AMR and poor health and sanitation (Hendriksen et al., 2019). Asian isolates were probably exposed to warmer temperatures than the European ones. A positive association between antibiotic resistance and the warm environmental temperature has been already shown in terrestrial animals and humans and the same association was recently found in the marine environment. A possible explanation is that warmer temperatures impair the immune response of fish to pathogens, making them more susceptible to infections and finally resulting in an increased need for antibiotic therapy (Reverter et al., 2020). In addition, warm temperatures promote the maintenance and spread of ARGs, as a possible mechanism of adaptation of the bacterial cell to the thermal stress (Van Boeckel et al., 2019; Rodríguez-Verdugo et al., 2020).

Our study has some limitations: first, studies were unevenly distributed in different regions and bacterial species, thus reducing the possibility to associate resistance levels to a given area. The majority of the studies were carried out in Asia or in Europe, whilst other continents, such as Africa, were severely underrepresented. Secondly, the quality assessment revealed that most studies were based on convenience sampling, and they might not be representative of the area of origin. In most cases, the geographical origin of samples was not reported. The methods used to assess AMR varied, particularly for marine bacteria, reflecting a lack of standardisation of susceptibility testing for these microorganisms, as already described by other authors (Baron et al., 2017; Lin et al., 2022; Yuan et al., 2022). In addition, the studies tested a range of different antibiotics, sometimes belonging to the same class, reporting only aggregate data. Such lack of information at the isolate level dramatically impairs the identification of multiple resistance in the same isolate, ultimately preventing the detection of high-risk clones. Where only aggregate data were available, we assumed that the number of isolates resistant to a class corresponded to the highest number of resistant isolates recorded for the same class. In these cases, we might have underestimated the number of isolates resistant to an antibiotic class. To avoid these limitations, we strongly suggest that primary studies report antibiotic-resistant data at the isolate level in the future. Despite these limitations, systematic reviews based on point prevalence studies have already provided a source of data for AMR in fish or in marine environments (Van Boeckel et al., 2019; Reverter et al., 2020; Onohuean et al., 2022). These reviews have been successfully used to estimate the prevalence of AMR in marine bacteria as well as to identify hotspots or risk factors for contamination, filling a data gap due to the lack of systematic surveillance programmes (Reverter et al., 2020; Onohuean et al., 2022).

In conclusion, we found elevated levels of antibiotic resistance to first-line antibiotics in bacteria isolates from marine bivalves, either of terrestrial or marine origin. In most cases, a moderate prevalence of resistance was found for last-resort antimicrobials, particularly in bacteria from the marine environment. Regardless of the bacterial genera, antibiotic resistance was higher in Asian than in European isolates. Since bivalves are often consumed raw or undercooked, the presence of a high prevalence rate of AMR in this type of food can be regarded as a risk for human health. Our findings also highlight the need for standardised surveillance of AMR in bivalves.

## Author contributions

CFM, FL, and FC: study conception. MO, FC, CFM, and FL: design of the study. EA, LS, MO, FC, FL, and CFM: literature search and data analysis. MO, EA, FL, and CFM: analysis of the results and drafting the manuscript. FL and CFM: manuscript final revision. All authors contributed to the article and approved the submitted version.

## Funding

This work was supported by the Italian Ministry of Health through project IZSUM RC004/2019 and regional funding through project RCIS12019 are acknowledged.

## Conflict of interest

The authors declare that the research was conducted in the absence of any commercial or financial relationships that could be construed as a potential conflict of interest.

## Publisher’s note

All claims expressed in this article are solely those of the authors and do not necessarily represent those of their affiliated organizations, or those of the publisher, the editors and the reviewers. Any product that may be evaluated in this article, or claim that may be made by its manufacturer, is not guaranteed or endorsed by the publisher.
