# Supplementary material for: A systematic review and meta-analysis on antimicrobial resistance in marine bivalves
Source: Front Microbiol. 2022 Dec 1;13:1040568. doi: 10.3389/fmicb.2022.1040568 (PMC9751792; doi:10.3389/fmicb.2022.1040568)
Supplement: Supplementary file 1 [file Table_1.DOCX]

**Appendix 1. Literature search strategies.**

| **Pubmed 14/04/2020** | | |
| --- | --- | --- |
| #1 | ("Bivalvia"[Mesh] OR seafood[Title/Abstract] OR bivalve OR bivalves OR mussel OR mussels OR clam OR clams OR oyster OR oysters OR ostrea edulis OR crassostrea OR scallop OR scallops OR pecten maximus OR chamelea gallina OR venus gallina OR venus verrucosa OR ruditapes decussatus OR filter-feeding organism OR filter-feeding organisms OR anadara OR cockle OR cockles OR mytella guyanensis OR mytilus OR "Perna"[Mesh] OR perna OR modiolus OR dreissena polymorpha OR mollusc OR molluscs OR mollusk OR mollusks OR molluscan shellfish OR aquatic invertebrate* OR ensis OR solen OR callista OR donax OR chlamys OR flexopecten proteus OR argopecten tehuelchus OR aequipecten OR arca noae OR meretrix OR ameghinomya antiqua OR paphia undulata OR transennella pannosa OR dosinia exoleta OR trivela mactroides OR venerupis decussata OR tapes semidecussatus OR mesodesma donacium) | 81,477 |
| #2 | "Drug Resistance, Microbial"[Mesh] OR ((antibacterial OR antimicrobial OR antibiotic OR drug OR multidrug) AND (resistance OR resistant)) OR ABR OR AMR OR MDR OR resistance genes OR horizontal gene transfer OR resistance determinants OR multi-resistance OR multiresistance OR antibiotic susceptibility OR resistome OR resistomes OR "Carbapenem-Resistant Enterobacteriaceae"[Mesh] OR "beta-Lactam Resistance"[Mesh] OR ((beta-lactam OR beta-lactams) AND (resistance OR resistant)) OR ((carbapenem OR carbapenems) AND (resistance OR resistant)) OR (extended-spectrum beta-lactamase) | 722,043 |
| #3 | "Enterobacteriaceae"[Mesh] OR enterobacteriaceae OR klebsiella oxytoca OR K. oxytoca OR klebsiella pneumoniae OR K. pneumoniae OR citrobacter freundii OR C. freundii OR edwardsiella ictaluri OR E. ictaluri OR edwardsiella tarda OR E. tarda OR enterobacter aerogenes OR E. aerogenes OR enterobacter cloacae OR E. cloacae OR cronobacter sakazakii OR C. sakazakii OR citrobacter freundii OR C. freundii OR citrobacter koseri OR C. koseri OR citrobacter rodentium OR C. rodentium OR erwinia amylovora OR E. amylovora OR hafnia alvei OR H. alvei OR morganella morganii OR M. morganii OR pantoea agglomerans OR P. agglomerans OR pectobacterium carotovorum OR P. carotovorum OR pectobacterium chrysanthemi OR P. chrysanthemi OR plesiomonas shigelloides OR P. shigelloides OR proteus mirabilis OR P. mirabilis OR proteus penneri OR P. penneri OR proteus vulgaris OR P. vulgaris OR Providencia sp. OR serratia marcescens OR S. marcescens OR serratia liquefaciens OR S. liquefaciens OR shigella boydii OR S. boydii OR shigella dysenteriae OR S. dysenteriae OR shigella flexneri OR S. flexneri OR shigella sonnei OR S. sonnei OR yersinia enterocolitica OR Y. enterocolitica OR yersinia pseudotuberculosis OR Y. pseudotuberculosis OR yersinia ruckeri OR Y. ruckeri OR escherichia coli OR E. Coli OR "Enterococcaceae"[Mesh] OR enterococci OR ((enterococcus OR E.) AND (faecalis OR faecium OR hirae OR casseliflavus OR avium OR durans OR spp)) OR "Vibrionaceae"[Mesh] OR ((vibrio OR v.) AND (spp OR parahaemolyticus OR vulnificus OR crassostreae OR alginolyticus OR metschnikovii OR coralliilyticus OR cholerae OR mimicus OR furnissii OR anguillarum OR campbellii OR fluvialis OR harveyi OR splendidus OR fischeri)) OR "Salmonella"[Mesh] OR salmonella OR "Aeromonadaceae"[Mesh] OR aeromonas spp OR aeromonad OR aeromonads OR aeromonas caviae OR A. caviae OR aeromonas hydrophila OR A. hydrophila OR aeromonas salmonicida OR A. salmonicida OR aeromonas veronii OR A. veronii OR ((arcobacter OR a.) AND (spp OR butzleri OR cryaerophilus OR skirrowii OR cloacae OR venerupis OR aquimarinus OR anaerophilus OR bivalviorum OR canalis OR cibarius OR ellissii OR defluvii OR ebronensis OR lanthierii OR halophilus OR molluscorum OR marinus OR mytili OR porcinus OR suis OR thereius OR trophiarum OR nitrofigilis OR pacificus)) OR arcobacters OR "Pseudomonadaceae"[Mesh] OR (Pseudomonas AND (spp OR aeruginosa OR alcaligenes OR chlororaphis OR fluorescens OR fragi OR mendocina OR oleovorans OR pseudoalcaligenes OR putida OR stutzeri OR syringae)) OR (burkholderia cepacia) OR (chryseobacterium indologenes) OR "Moraxellaceae"[Mesh] OR moraxella catarrhalis OR M. catarrhalis OR moraxella bovis OR M. bovis OR (Acinetobacter AND (spp OR baumannii OR calcoaceticus)) OR "Clostridium"[Mesh] OR clostridium botulinum OR C. botulinum OR clostridium difficile OR C. difficile OR clostridium perfringens OR C. perfringens OR clostridium sordellii OR C. sordellii OR clostridium kluyveri OR C. kluyveri OR clostridium histolyticum OR C. hystoliticum OR clostrium butyricum OR C. butyricum OR "Staphylococcus"[Mesh] OR ((staphylococcus OR S.) AND (aureus OR capitis OR epidermidis OR haemolyticus OR hominis OR hyicus OR intermedius OR lugdunensis OR saprophyticus)) OR "Shewanella"[Mesh] OR shewanella putrefaciens OR S. putrefaciens OR shewanella haliotis OR S. haliotis OR "Campylobacter"[Mesh] OR ((campylobacter OR c.) AND (spp OR coli OR jejuni OR fetus OR hyointestinalis OR lari OR rectus OR upsaliensis OR sputorum OR peloridis)) | 886,275 |
| #4 | (#1 AND #2 AND #3) Filters: Italian; English | 485 |
|  | **Update 03/09/2021** | 132 |

| **Embase 14/04/2020** | | |
| --- | --- | --- |
| #1 | 'bivalve'/exp OR 'bivalve' OR 'bivalves' OR 'bivalvia' OR 'sea food':ti,ab,kw OR 'seafood':ti,ab,kw OR mussel OR mussels OR clam OR clams OR oyster OR oysters OR 'ostrea edulis' OR crassostrea OR scallop OR scallops OR 'pecten maximus' OR 'chamelea gallina' OR 'venus gallina' OR 'venus verrucosa' OR 'ruditapes decussatus' OR 'filter-feeding organism' OR 'filter-feeding organisms' OR anadara OR cockle OR cockles OR 'mytella guyanensis' OR mytilus OR 'perna'/exp OR perna OR modiolus OR 'dreissena polymorpha' OR mollusc OR molluscs OR mollusk OR mollusks OR 'molluscan shellfish' OR 'aquatic invertebrate*' OR ensis OR solen OR callista OR donax OR chlamys OR 'flexopecten proteus' OR 'argopecten tehuelchus' OR aequipecten OR 'arca noae' OR meretrix OR 'ameghinomya antiqua' OR 'paphia undulata' OR 'transennella pannosa' OR 'dosinia exoleta' OR 'trivela mactroides' OR 'venerupis decussata' OR 'tapes semidecussatus' OR 'mesodesma donacium' | 62,591 |
| #2 | 'antibiotic resistance' OR 'antibiotic resistance'/exp OR 'multidrug resistance' OR 'multidrug resistance'/exp OR ((antibacterial OR antimicrobial OR antibiotic OR drug OR multidrug) AND (resistance OR resistant)) OR abr OR amr OR mdr OR 'resistance genes' OR 'horizontal gene transfer' OR 'resistance determinants' OR 'multi-resistance' OR multiresistance OR 'antibiotic susceptibility' OR resistome OR resistomes OR 'carbapenem-resistant enterobacteriaceae' OR 'carbapenem-resistant enterobacteriaceae'/exp OR 'beta-lactam resistance' OR 'beta-lactam resistance'/exp OR (('beta lactam' OR 'beta lactams') AND (resistance OR resistant)) OR ((carbapenem OR carbapenems) AND (resistance OR resistant)) OR ('extended spectrum' AND 'beta lactamase') | 983,348 |
| #3 | 'enterobacteriaceae' OR 'enterobacteriaceae'/exp OR 'klebsiella oxytoca' OR 'k. oxytoca' OR 'klebsiella pneumoniae' OR 'k. pneumoniae' OR 'edwardsiella ictaluri' OR 'e. ictaluri' OR 'edwardsiella tarda' OR 'e. tarda' OR 'enterobacter aerogenes' OR 'e. aerogenes' OR 'enterobacter cloacae' OR 'e. cloacae' OR 'cronobacter sakazakii' OR 'c. sakazakii' OR 'citrobacter freundii' OR 'c. freundii' OR 'citrobacter koseri' OR 'c. koseri' OR 'citrobacter rodentium' OR 'c. rodentium' OR 'erwinia amylovora' OR 'e. amylovora' OR 'hafnia alvei' OR 'h. alvei' OR 'morganella morganii' OR 'm. morganii' OR 'pantoea agglomerans' OR 'p. agglomerans' OR 'pectobacterium carotovorum' OR 'p. carotovorum' OR 'pectobacterium chrysanthemi' OR 'p. chrysanthemi' OR 'plesiomonas shigelloides' OR 'p. shigelloides' OR 'proteus mirabilis' OR 'p. mirabilis' OR 'proteus penneri' OR 'p. penneri' OR 'proteus vulgaris' OR 'p. vulgaris' OR 'providencia sp.' OR 'serratia marcescens' OR 's. marcescens' OR 'serratia liquefaciens' OR 's. liquefaciens' OR 'shigella boydii' OR 's. boydii' OR 'shigella dysenteriae' OR 's. dysenteriae' OR 'shigella flexneri' OR 's. flexneri' OR 'shigella sonnei' OR 's. sonnei' OR 'yersinia enterocolitica' OR 'y. enterocolitica' OR 'yersinia pseudotuberculosis' OR 'y. pseudotuberculosis' OR 'yersinia ruckeri' OR 'y. ruckeri' OR 'escherichia coli' OR 'e. coli' OR 'enterococcaceae' OR 'enterococcaceae'/exp OR enterococci OR ((enterococcus OR e.) AND (faecalis OR faecium OR hirae OR casseliflavus OR avium OR durans OR spp)) OR 'vibrionaceae' OR 'vibrionaceae'/exp OR ((vibrio OR v.) AND (spp OR parahaemolyticus OR vulnificus OR crassostreae OR alginolyticus OR metschnikovii OR coralliilyticus OR cholerae OR mimicus OR furnissii OR anguillarum OR campbellii OR fluvialis OR harveyi OR splendidus OR fischeri)) OR 'salmonella' OR 'salmonella'/exp OR 'aeromonadaceae' OR 'aeromonadaceae'/exp OR 'aeromonas spp' OR aeromonad OR aeromonads OR 'aeromonas caviae' OR 'a. caviae' OR 'aeromonas hydrophila' OR 'a. hydrophila' OR 'aeromonas salmonicida' OR 'a. salmonicida' OR 'aeromonas veronii' OR 'a. veronii' OR ((arcobacter OR a.) AND (spp OR butzleri OR cryaerophilus OR skirrowii OR cloacae OR venerupis OR aquimarinus OR anaerophilus OR bivalviorum OR canalis OR cibarius OR ellissii OR defluvii OR ebronensis OR lanthierii OR halophilus OR molluscorum OR marinus OR mytili OR porcinus OR suis OR thereius OR trophiarum OR nitrofigilis OR pacificus)) OR arcobacters OR 'pseudomonadaceae' OR 'pseudomonadaceae'/exp OR (pseudomonas AND (spp OR aeruginosa OR alcaligenes OR chlororaphis OR fluorescens OR fragi OR mendocina OR oleovorans OR pseudoalcaligenes OR putida OR stutzeri OR syringae)) OR 'burkholderia cepacia' OR 'chryseobacterium indologenes' OR 'moraxellaceae' OR 'moraxellaceae'/exp OR 'moraxella catarrhalis' OR 'm. catarrhalis' OR 'moraxella bovis' OR 'm. bovis' OR (acinetobacter AND (spp OR baumannii OR calcoaceticus)) OR 'clostridium' OR 'clostridium'/exp OR 'clostridium botulinum' OR 'c. botulinum' OR 'clostridium difficile' OR 'c. difficile' OR 'clostridium perfringens' OR 'c. perfringens' OR 'clostridium sordellii' OR 'c. sordellii' OR 'clostridium kluyveri' OR 'c. kluyveri' OR 'clostridium histolyticum' OR 'c. hystoliticum' OR 'clostrium butyricum' OR 'c. butyricum' OR 'staphylococcus' OR 'staphylococcus'/exp OR ((staphylococcus OR s.) AND (aureus OR capitis OR epidermidis OR haemolyticus OR hominis OR hyicus OR intermedius OR lugdunensis OR saprophyticus)) OR 'shewanella' OR 'shewanella'/exp OR 'shewanella putrefaciens' OR 's. putrefaciens' OR 'shewanella haliotis' OR 's. haliotis' OR 'campylobacter' OR 'campylobacter'/exp OR ((campylobacter OR c.) AND (spp OR coli OR jejuni OR fetus OR hyointestinalis OR lari OR rectus OR upsaliensis OR sputorum OR peloridis)) | 1,281,686 |
| #4 | #1 AND #2 AND #3 | 554 |
| #5 | #4 AND ([english]/lim OR [italian]/lim) | 522 |
|  | **Update 03/09/2021** | 161 |

| **Web of Science 14/04/2020** | | |
| --- | --- | --- |
| #1 | TS=(bivalvia OR seafood OR bivalve OR bivalves OR mussel OR mussels OR clam OR clams OR oyster OR oysters OR "ostrea edulis" OR crassostrea OR scallop OR scallops OR "pecten maximus" OR "chamelea gallina" OR "venus gallina" OR "venus verrucosa" OR "ruditapes decussatus" OR "filter-feeding organism" OR "filter-feeding organisms" OR anadara OR cockle OR cockles OR "mytella guyanensis" OR mytilus OR perna OR modiolus OR "dreissena polymorpha" OR mollusc OR molluscs OR mollusk OR mollusks OR "molluscan shellfish" OR "aquatic invertebrate*" OR ensis OR solen OR callista OR donax OR chlamys OR "flexopecten proteus" OR "argopecten tehuelchus" OR aequipecten OR "arca noae" OR meretrix OR "ameghinomya antiqua" OR "paphia undulata" OR "transennella pannosa" OR "dosinia exoleta" OR "trivela mactroides" OR "venerupis decussata" OR "tapes semidecussatus" OR "mesodesma donacium")  Indexes=SCI-EXPANDED, SSCI, A&HCI, CPCI-S, CPCI-SSH, ESCI Timespan=All years | 114,346 |
| #2 | TS=("antibiotic resistance" OR "antimicrobial resistance" OR "antibacterial resistance" OR "multidrug resistance" OR ((antibacterial OR antimicrobial OR antibiotic OR drug OR multidrug) AND (resistance OR resistant)) OR ABR OR AMR OR MDR OR "resistance genes" OR "horizontal gene transfer" OR "resistance determinants" OR "multi-resistance" OR multiresistance OR "antibiotic susceptibility" OR resistome OR resistomes OR "carbapenem-resistant enterobacteriaceae" OR "beta-lactam resistance" OR (("beta lactam" OR "beta lactams") AND (resistance OR resistant)) OR ((carbapenem OR carbapenems) AND (resistance OR resistant)) OR ("extended spectrum" AND "beta lactamase"))  Indexes=SCI-EXPANDED, SSCI, A&HCI, CPCI-S, CPCI-SSH, ESCI Timespan=All years | 424,266 |
| #3 | TS=("enterobacteriaceae" OR "klebsiella oxytoca" OR "k. oxytoca" OR "klebsiella pneumoniae" OR "k. pneumoniae" OR "edwardsiella ictaluri" OR "e. ictaluri" OR "edwardsiella tarda" OR "e. tarda" OR "enterobacter aerogenes" OR "e. aerogenes" OR "enterobacter cloacae" OR "e. cloacae" OR "cronobacter sakazakii" OR "c. sakazakii" OR "citrobacter freundii" OR "c. freundii" OR "citrobacter koseri" OR "c. koseri" OR “citrobacter rodentium” OR “c. rodentium” OR “erwinia amylovora” OR “e. amylovora” OR “hafnia alvei” OR “h. alvei” OR “morganella morganii” OR “m. morganii” OR “pantoea agglomerans” OR “p. agglomerans” OR “pectobacterium carotovorum” OR “p. carotovorum” OR “pectobacterium chrysanthemi” OR “p. chrysanthemi” OR “plesiomonas shigelloides” OR “p. shigelloides” OR “proteus mirabilis” OR “p. mirabilis” OR “proteus penneri” OR “p. penneri” OR “proteus vulgaris” OR “p. vulgaris” OR “providencia sp.” OR “serratia marcescens” OR “s. marcescens” OR “serratia liquefaciens” OR “s. liquefaciens” OR “shigella boydii” OR “s. boydii” OR “shigella dysenteriae” OR “s. dysenteriae” OR “shigella flexneri” OR “s. flexneri” OR “shigella sonnei” OR “s. sonnei” OR “yersinia enterocolitica” OR “y. enterocolitica” OR “yersinia pseudotuberculosis” OR “y. pseudotuberculosis” OR “yersinia ruckeri” OR “y. ruckeri” OR “escherichia coli” OR “e. coli” OR “enterococcaceae” OR enterococci OR ((enterococcus OR e.) AND (faecalis OR faecium OR hirae OR casseliflavus OR avium OR durans OR spp)) OR “vibrionaceae” OR (vibrio OR v.) AND (spp OR parahaemolyticus OR vulnificus OR crassostreae OR alginolyticus OR metschnikovii OR coralliilyticus OR cholerae OR mimicus OR furnissii OR anguillarum OR campbellii OR fluvialis OR harveyi OR splendidus OR fischeri) OR “salmonella” OR “aeromonadaceae” OR “aeromonas spp” OR aeromonad OR aeromonads OR “aeromonas caviae” OR “a. caviae” OR “aeromonas hydrophila” OR “a. hydrophila” OR “aeromonas salmonicida” OR “a. salmonicida” OR “aeromonas veronii” OR “a. veronii” OR ((arcobacter OR a.) AND (spp OR butzleri OR cryaerophilus OR skirrowii OR cloacae OR venerupis OR aquimarinus OR anaerophilus OR bivalviorum OR canalis OR cibarius OR ellissii OR defluvii OR ebronensis OR lanthierii OR halophilus OR molluscorum OR marinus OR mytili OR porcinus OR suis OR thereius OR trophiarum OR nitrofigilis OR pacificus)) OR arcobacters OR “pseudomonadaceae” OR pseudomonas AND (spp OR aeruginosa OR alcaligenes OR chlororaphis OR fluorescens OR fragi OR mendocina OR oleovorans OR pseudoalcaligenes OR putida OR stutzeri OR syringae) OR “burkholderia cepacia” OR “chryseobacterium indologenes” OR “moraxellaceae” OR “moraxella catarrhalis” OR “m. catarrhalis” OR “moraxella bovis” OR “m. bovis” OR (acinetobacter AND (spp OR baumannii OR calcoaceticus)) OR “clostridium” OR “clostridium botulinum” OR “c. botulinum” OR “clostridium difficile” OR “c. difficile” OR “clostridium perfringens” OR “c. perfringens” OR “clostridium sordellii” OR “c. sordellii” OR “clostridium kluyveri” OR “c. kluyveri” OR “clostridium histolyticum” OR “c. hystoliticum” OR “clostrium butyricum” OR “c. butyricum” OR “staphylococcus” OR (staphylococcus OR s.) AND (aureus OR capitis OR epidermidis OR haemolyticus OR hominis OR hyicus OR intermedius OR lugdunensis OR saprophyticus) OR “shewanella” OR “shewanella putrefaciens” OR “s. putrefaciens” OR “shewanella haliotis” OR “s. haliotis” OR “campylobacter” OR (campylobacter OR c.) AND (spp OR coli OR jejuni OR fetus OR hyointestinalis OR lari OR rectus OR upsaliensis OR sputorum OR peloridis))  Indexes=SCI-EXPANDED, SSCI, A&HCI, CPCI-S, CPCI-SSH, ESCI Timespan=All years | 1,011,440 |
| #4 | (#1 AND #2 AND #3)  Indexes=SCI-EXPANDED, SSCI, A&HCI, CPCI-S, CPCI-SSH, ESCI Timespan=All years Refined by: LANGUAGES: (ENGLISH) | 593 |
|  | **Update 03/09/2021** | 162 |
