# Supplementary material for: A systematic review and meta-analysis on antimicrobial resistance in marine bivalves
Source: Front Microbiol. 2022 Dec 1;13:1040568. doi: 10.3389/fmicb.2022.1040568 (PMC9751792; doi:10.3389/fmicb.2022.1040568)
Supplement: Supplementary file 2 [file Table_2.DOCX]

**Appendix 2. Full-text articles excluded and reasons for exclusion.**

|  | **References** | **Reasons for exclusion** |
| --- | --- | --- |
| 1 | Álvarez-Contreras 2021 | unclear data of AMR |
| 2 | Campista-León 2021 | study not on bivalves |
| 3 | Chen 2021 | unclear data of AMR |
| 4 | Chen 2021 | unclear source of isolates |
| 5 | Giacometti 2021 | unclear data of AMR |
| 6 | Jingjit 2021 | unclear data of AMR |
| 7 | Kukułowicz 2021 | not a study on AMR |
| 8 | Kurittu 2021 | no isolates from bivalves |
| 9 | La Tela 2021 | dead and moribund bivalves |
| 10 | Lattos 2021 | dead and moribund bivalves |
| 11 | Menon 2021 | unclear data of AMR |
| 12 | Sadat 2021 | unclear data of AMR |
| 13 | Sánchez 2021 | insufficient description of sampling |
| 14 | Wu 2021 | study not on bivalves |
| 15 | Citterio 2020 | unclear data of AMR |
| 16 | Fu 2020 | unclear data of AMR |
| 17 | Håkonsholm 2020 | unclear data of AMR |
| 18 | Hu 2020 | unclear data of AMR |
| 19 | Karp 2020 | study not on bivalves |
| 20 | Kim 2020 | study not on bivalves |
| 21 | Kotian 2020 | unclear data of AMR |
| 22 | Kotian 2020 | not a study on AMR |
| 23 | Oliveira 2020 | unclear data of AMR |
| 24 | Saha 2020 | study not on bivalves |
| 25 | Singh 2020 | study not on bivalves |
| 26 | Su 2020 | unclear data of AMR |
| 27 | Taha 2020 | unclear data of AMR |
| 28 | Tan 2020 | unclear data of AMR |
| 29 | Osawa 2020 | insufficient description of sampling |
| 30 | Kim 2020 | insufficient description of sampling |
| 31 | Wickramanayake 2020 | study not on bivalves |
| 32 | Kernéis 2020 | not a prevalence study |
| 33 | Narayanan 2020 | unclear source of isolates |
| 34 | Brouwer 2019 | study not on bivalves |
| 35 | Mok 2019 | unclear source of isolates |
| 36 | Naas 2019 | not a study on AMR |
| 37 | On 2019 | not a study on AMR |
| 38 | Wu 2019 | study not on bivalves |
| 39 | Reshma 2019 | insufficient description of sampling |
| 40 | Jeamsripong 2019 | not a study on AMR |
| 41 | Brouwer 2019 | study not on bivalves |
| 42 | Fang 2019 | unclear source of isolates |
| 43 | Pan 2019 | insufficient description of sampling |
| 44 | Das, Uday Narayan 2019 | unclear source of isolates |
| 45 | Lee 2019 | insufficient description of sampling |
| 46 | Balbi 2019 | not a study on AMR |
| 47 | Bighiu 2019 | interventional study |
| 48 | Mok 2019 | unclear source of isolates |
| 49 | Basha 2019 | study not on bivalves |
| 50 | Nayebpour 2019 | article retracted |
| 51 | Baliga, Pallavi 2019 | unclear source of isolates |
| 52 | Pramono 2019 | unclear source of isolates |
| 53 | Terzi 2019 | unclear data of AMR |
| 54 | Jiang 2019 | unclear source of isolates |
| 55 | Samayita 2018 | unclear data of AMR |
| 56 | Mani 2018 | not a study on AMR |
| 57 | Tran 2018 | unclear source of isolates |
| 58 | Harada 2018 | unclear source of isolates |
| 59 | Briet 2018 | study not on bivalves |
| 60 | Banerjee 2018 | not a study on AMR |
| 61 | Yamaguchi 2018 | study not on bivalves |
| 62 | Ottaviani 2018 | unclear source of isolates |
| 63 | Li 2018 | study not on bivalves |
| 64 | Elmahdi 2018 | interventional study |
| 65 | Oranusi 2018 | unclear source of isolates |
| 66 | Sharafati Chaleshtori 2018 | study not on bivalves |
| 67 | Mastrodonato 2018 | study not on bivalves |
| 68 | Terzi, Ertugrul | unclear source of isolates |
| 69 | Cardoso 2018 | unclear source of isolates |
| 70 | Roschanski 2017 | unclear data of AMR |
| 71 | Said 2017 | study not on bivalves |
| 72 | Rathlavath 2017 | study not on bivalves |
| 73 | Mechri 2017 | dead and moribund bivalves |
| 74 | Sanjit Singh 2017 | unclear source of isolates |
| 75 | Eggermont 2017 | challenge study |
| 76 | Fernandez-Delgado 2017 | unclear source of isolates |
| 77 | Brandao 2017 | unclear data of AMR |
| 78 | Murugadas 2017 | not a study on AMR |
| 79 | Grevskott 2017 | unclear data of AMR |
| 80 | Morejon 2017 | unclear data of AMR |
| 81 | Economopoulou 2017 | study not on bivalves |
| 82 | Citterio 2017 | unclear data of AMR |
| 83 | Rodriguez-Souto 2017 | insufficient description of sampling |
| 84 | Romero 2017 | unclear source of isolates |
| 85 | Sivaraman 2017 | study not on bivalves |
| 86 | Naas 2017 | study not on bivalves |
| 87 | Ben Said 2017 | insufficient description of sampling |
| 88 | Abdella 2017 | challenge study |
| 89 | Sivaraman 2017 | study not on bivalves |
| 90 | Hernandez-Robles 2016 | unclear data of AMR |
| 91 | Dubert 2016 | challenge study |
| 92 | Stark 2016 | not a study on AMR |
| 93 | Boss 2016 | unclear source of isolates |
| 94 | Guo 2016 | study not on bivalves |
| 95 | Rubini 2016 | insufficient description of sampling |
| 96 | Abdollahzadeh 2016 | study not on bivalves |
| 97 | Jayme 2016 | unclear source of isolates |
| 98 | Xie 2016 | study not on bivalves |
| 99 | Xu 2016 | unclear source of isolates |
| 100 | Murugadas 2016 | study not on bivalves |
| 101 | Deekshit 2016 | study not on bivalves |
| 102 | Murugadas 2016 | unclear source of isolates |
| 103 | da Silveira 2016 | unclear source of isolates |
| 104 | Garrido-Maestu 2016 | unclear data of AMR |
| 105 | Deekshit 2015 | study not on bivalves |
| 106 | Miranda 2015 | challenge study |
| 107 | Li 2015 | study not on bivalves |
| 108 | Ceccarelli 2015 | unclear source of isolates |
| 109 | Ahmed 2015 | study not on bivalves |
| 110 | Mechri 2015 | dead and moribund bivalves |
| 111 | Preeprem 2014 | unclear source of isolates |
| 112 | Yu 2014 | study not on bivalves |
| 113 | Wang 2014 | unclear source of isolates |
| 114 | Fajardo 2014 | interventional study |
| 115 | Ananchaipattana 2014 | unclear source of isolates |
| 116 | Sudha 2014 | unclear source of isolates |
| 117 | Ahmad 2014 | insufficient description of sampling |
| 118 | Yu 2014 | unclear source of isolates |
| 119 | Elexson 2014 | unclear source of isolates |
| 120 | Mechri 2013 | study not on bivalves |
| 121 | Liang 2013 | study not on bivalves |
| 122 | Kumar 2013 | unclear source of isolates |
| 123 | Maravic 2013b | unclear source of isolates |
| 124 | Miranda 2013 | challenge study |
| 125 | Selim 2013 | unclear source of isolates |
| 126 | Wei 2013 | unclear data of AMR |
| 127 | Malainine 2013 | unclear source of isolates |
| 128 | Deekshit 2012 | unclear source of isolates |
| 129 | Maravic 2012 | unclear data of AMR |
| 130 | Woodring 2012 | unclear source of isolates |
| 131 | Jun 2012 | unclear source of isolates |
| 132 | Akiyama 2012 | study not on bivalves |
| 133 | Ryu 2012 | unclear source of isolates |
| 134 | Raissy 2012 | study not on bivalves |
| 135 | Wang 2012 | study not on bivalves |
| 136 | Mechri 2012 | unclear source of isolates |
| 137 | Meng 2011 | study not on bivalves |
| 138 | Akiyama 2011 | study not on bivalves |
| 139 | Lagana 2011 | unclear data of AMR |
| 140 | Al-Othrubi 2011 | unclear source of isolates |
| 141 | Yan 2010 | study not on bivalves |
| 142 | Kumaran 2010 | study not on bivalves |
| 143 | Mejdi 2010 | unclear source of isolates |
| 144 | Alagarsamy 2010 | study not on bivalves |
| 145 | Stonsaovapak 2010 | study not on bivalves |
| 146 | Setti 2009 | unclear source of isolates |
| 147 | Bouchrif 2009 | study not on bivalves |
| 148 | Chao 2009 | unclear source of isolates |
| 149 | Daramola 2009 | unclear source of isolates |
| 150 | Kumar 2009a | study not on bivalves |
| 151 | Kumar 2009b | study not on bivalves |
| 152 | Le Roux 2009 | dead and moribund bivalves |
| 153 | Khan 2009 | unclear source of isolates |
| 154 | Baker-Austin 2009 | sediment and water sample |
| 155 | Fernandez-Delgado 2009 | unclear source of isolates |
| 156 | Vongxay 2008 | unclear source of isolates |
| 157 | Rahmati 2008 | unclear source of isolates |
| 158 | Adeyemi 2008 | study not on bivalves |
| 159 | Chakraborty 2008 | study not on bivalves |
| 160 | Ferrini 2008 | unclear source of isolates |
| 161 | Beleneva 2008 | unclear source of isolates |
| 162 | Valdezate 2007 | unclear source of isolates |
| 163 | Marlina 2007 | unclear data of AMR |
| 164 | Bourouni 2007 | unclear data of AMR |
| 165 | Khan 2006 | unclear source of isolates |
| 166 | Martinez-Urtaza 2005 | processed mussels |
| 167 | Kumar 2005 | unclear source of isolates |
| 168 | OzFoodNet Working Group 2004 | not a study on AMR |
| 169 | Terajima 2004 | unclear source of isolates |
| 170 | Balotescu 2003 | unclear source of isolates |
| 171 | Zhao 2003 | not a study on AMR |
| 172 | Ottaviani 2001 | unclear source of isolates |
| 173 | Tzelepi 1999 | unclear source of isolates |
| 174 | Riquelme 1996 | study not on bivalves |
| 175 | Singh 1994 | unclear source of isolates |
| 176 | Singh 1993 | unclear source of isolates |
| 177 | Castro 1992 | unclear data of AMR |
| 178 | Paille 1987 | unclear data of AMR |
| 179 | Molitoris 1985 | study not on bivalves |
| 180 | DiSalvo 1978 | not a study on AMR |
| 181 | Cooke 1976 | unclear data of AMR |
