# Supplementary material for: A systematic review and meta-analysis on antimicrobial resistance in marine bivalves
Source: Front Microbiol. 2022 Dec 1;13:1040568. doi: 10.3389/fmicb.2022.1040568 (PMC9751792; doi:10.3389/fmicb.2022.1040568)
Supplement: Supplementary file 3 [file Table_3.DOCX]

**Appendix 3. Antibiotics list**

| **Class** | **Antibiotic** |
| --- | --- |
| Penicillins | Ampicillin  Piperacillin  Amoxicillin  Penicillin  Oxacillin  Mecillinam |
| Tetracyclines | Tetracycline  Oxytetracycline  Doxycycline  Chlortetracycline  Minocycline |
| Polymyxin | Colistin  Polimixina B |
| Amphenicols | Cloramphenicol  Florfenicol |
| Aminoglycosides | Streptomycin  Gentamicin  Kanamycin  Amikacin  Tobramycin  Netilmicin  Neomycin  Isepamicin  Apramycin |
| Macrolides | Erythromycin  Azithromycin  Clarithromycin  Midecamycin |
| Quinolones | Nalidixic acid  Oxolinic acid |
| Fluoroquinolones | Ciprofloxacin  Ofloxacin  Moxifloxacin  Norfloxacin  Levofloxacin  Enrofloxacin  Pefloxacin  Gatifloxacin  Lomefloxacin |
| Carbapenems | Imipenem  Meropenem  Ertapenem  Doripenem  Tebipenem |
| Trimethoprim - sulfonamide combinations | Trimethoprim-sulfamethoxazole  Cotrimoxazole |
| Rifamycins | Rifampicin |
| First/Second generation cephalosporins | Cephalothin  Cefoxitin  Cefuroxime  Cefazolin  Cefotetan  Cefadroxil  Cefamandole  Cephalexin  Cefradine |
| Third/Fourth/Fifth generation cephalosporins | Cefotaxime  Ceftriaxone  Cefoperazone  Cefditoren  Ceftazidime  Cefepime  Ceftiofur  Cefpodoxime  Moxalactam  Ceftaroline  Cefixime  Ceftizoxime |
| Lincosamides | Clindamycin  Lincomycin |
| Glycopeptides | Vancomycin  Teicoplanin |
| Oxazolidinones | Linezolid |
| Streptogramins | Quinupristin-dalfopristin |
| Polypeptide | Bacitracin |
| Aminocoumarin | Novobiocin |
| Beta lactam - beta lactamase inhibitor | Amoxicillin + Clavulanic acid  Piperacillin + Tazobactam  Ampicillin + Sulbactam  Ticarcillin + Clavulanic acid  Ceftazidime + Clavulanic acid  Cefotaxime + Clavulanic acid  Cephalosporin + Clavulanic acid |
| Phosphonics | Phosphomycin |
| Nitrofurans | Nitrofurantoin  Furazolidone |
| Glycylcyclines | Tigecyclin |
| Imidazoles | Metronidazole |
| Carboxypenicillins | Carbenicillin  Ticarcillin |
| Sulphonamides | Sulfonamide  Sulfisoxazole  Sulphafurazole  Sulfamethoxazole  Sulfadiazine |
| Trimethoprim | Trimethoprim |
| Monobactams | Aztreonam |
| Aminocyclitols | Spectinomycin |
| Macrocyclic | Fidaxomicin |
| Peptide | LFF571 |
