# Supplementary material for: A systematic review and meta-analysis on antimicrobial resistance in marine bivalves
Source: Front Microbiol. 2022 Dec 1;13:1040568. doi: 10.3389/fmicb.2022.1040568 (PMC9751792; doi:10.3389/fmicb.2022.1040568)
Supplement: Supplementary file 4 [file Table_4.DOCX]

**Appendix 4. Characteristics of included studies.**

| **Study ID** | **Type of bivalve** | **Area of origin of the samples** | **Bivalves collected from retails or production areas** | **Period of samples collection** | **Type of bacteria (genus)** | **N of isolates tested** | **MAR index** | **Type of test used for antibiotic susceptibility testing** | **Antibiotic class tested** |
| --- | --- | --- | --- | --- | --- | --- | --- | --- | --- |
| Zacharias 2021  doi:10.3390/antibiotics10050571 | Mussels | Germany | Environment | NA | Escherichia | 1 | 0.50 | MIC | FQ; C; 3-4-5 GC; βLI |
|  | Mussels | Germany | Environment | NA | Acinetobacter | 11 | 0.27 | MIC | FQ; C; 3-4-5 GC; βLI |
| Song 2021  doi:10.1016/j.aquaculture.2020.736043 | Clams | China | Aquaculture | 2018 | Vibrio | 1 | 0.62 | Disc diffusion test | P; TET; PM; AP; AM; M; FQ; SXT; 1-2 GC; L; GP; N; CP |
| Shahimi 2021  doi:10.1007/s11356-021-13665-4 | Cockles | Malaysia | Environment | June 2019 | Vibrio | 24 | 0.08 | Disc diffusion test | P; TET; AP; AM; M; GP; S |
| Parthasarathy 2021  doi:10.1007/s11274-021-03113-3 | Oysters | India | Environment | July 2014 to March 2015 and July 2017 to May 2018 | Vibrio | 10 | 0.59 | Disc diffusion test | P; TET; AP; AM; Q; FQ; 3-4-5 GC; T |
| Lozano-León 2021  doi:10.1016/j.mran.2021.100176 | Mussels | Spain | Aquaculture | February to June 2015 | Campylobacter | 7 | 0.43 | E-test | P; TET; AP; AM; M; FQ; C; SXT; 1-2 GC; L |
| Lee 2021  doi:10.1111/jam.14865 | Oysters, Scallops | Norway | Environment | June to October 2020 | Aeromonas | 22 | 0.45 | Disc diffusion test | P; TET; AM; M; Q; FQ; C; SXT; 3-4-5 GC |
| Jeong 2021  doi:10.1007/s10661-021-09201-z | Oysters | Republic of Korea | Environment/Aquaculture | October, December 2018 and April 2019 | Escherichia | 76 | 0.06 | Disc diffusion test | P; TET; AP; AM; Q; FQ; C; SXT; 3-4-5 GC; 1-2 GC; βLI; T; MO |
| Hsu 2021  doi:10.1016/j.scitotenv.2020.143665 | Oysters, Clams | Taiwan | Environment/Aquaculture | January 2016 to February 2017 | Bacillus | 3 | 0.30 | MIC | P; TET; AP; AM; M; FQ; C; SXT; R; L; GP |
| Hossain 2021  doi:10.1089/mdr.2020.0590 | Mussels | South Korea | Environment/Aquaculture | January 2018 to March 2019 | Aeromonas | 33 | 0.32 | Disc diffusion test | P; TET; AP; AM; Q; FQ; C; SXT; R; 3-4-5 GC; 1-2 GC |
| Zangoei-Fard 2020  doi:10.21608/ejvs.2020.26304.1164 | Oysters, Shellfish | Iran | Environment | October 2017 to October 2018 | Vibrio | 25 | 0.44 | Disc diffusion test | P; TET; AM; M; Q; FQ; SXT; 3-4-5 GC; ; 1-2 GC; GP |
| Yu 2020  doi:10.3844/ajbbsp.2020.568.578 | Oysters | China | Retail | July and August 2019 | Escherichia | 44 | 0.30 | Disc diffusion test | P; TET; PM; AP; AM; M; FQ; C; SXT; 3-4-5 GC; 1-2 GC; N; S |
| Peruzy 2020  doi:10.3390/antibiotics9070365 | Molluscan shellfish | Italy | NA | 2015 to 2019 | Salmonella | 48 | 0.14 | Disc diffusion test | P; TET; PM; AP; AM; Q; FQ; SXT; 3-4-5 GC |
| Jo 2020  doi:10.1016/j.marpolbul.2020.111246 | Oysters, Clams | Korea | Aquaculture | May to November 2018 | Vibrio | 38 | 0.60 | Disc diffusion test | P; TET; AP; AM; M; Q; FQ; SXT; R; 3-4-5 GC; 1-2 GC; GP |
| Jeamsripong 2020  doi:10.1093/femsec/fiaa081 | Oysters | Thailand | Aquaculture | March 2016 to February 2017 | Vibrio | 361 | 0.16 | MIC | P; TET; AP; AM; M; FQ; S; T |
| Hong To 2020  doi:10.3390/biology9100312. | Clams, Cockles | Vietnam | Retail/Aquaculture | 2016 and 2017 | Vibrio | 2 | 0.25 | Disc diffusion test | P; TET; PM; AP; AM; Q; FQ; C |
| Håkonsholm 2020  doi:10.3390/microorganisms8121909. | Mussels, Oysters, Scallops, Cockles, Carpet shells | Norway | Aquaculture/Environment | 2016, September 2019 to March 2020 | Klebsiella | 153 | 0.08 | Disc diffusion test | P; TET; AP; AM; FQ; C; SXT; 3-4-5 GC; 1-2 GC; βLI; N; GC; MO |
|  | Mussels, Oysters, Scallops, Cockles, Carpet shells | Norway | Aquaculture/Environment | 2016, September 2019 to March 2020 | Raoultella | 51 | 0.07 | Disc diffusion test | P; TET; AP; AM; FQ; C; SXT; 3-4-5 GC; 1-2 GC; βLI; N; GC; MO |
| Divya 2020  doi:10.1111/jfpp.14837 | Clams | India | Environment | 2011 to 2013 | Escherichia | 48 | 0.05 | Disc diffusion test | P; TET; AP; AM; Q; FQ; C; SXT; 3-4-5 GC; 1-2 GC; T |
| Dahanayake 2020  doi:10.1111/lam.13261 | Cockles | Korea | Retail | January to May 2018 | Aeromonas | 32 | 0.30 | Disc diffusion test | P; TET; PM; AP; AM; M; Q; FQ; C; SXT; R; 3-4-5 GC; 1-2 GC |
| Sugawara 2019  doi:10.1038/s41598-019-51002-5 | Clams | Vietnam | Retail | November to December 2017 | Escherichia | 1 | 0.67 | MIC | TET; PM; AM; FQ; C; FOS |
| Dahanayake 2019_a  doi:10.1111/lam.13232 | Cockles | Korea | Retail | January to May 2018 | Vibrio | 32 | 0.51 | Disc diffusion test | P; TET; PM; AP; AM; M; Q; FQ; C; SXT; R; 3-4-5 GC; 1-2 GC |
| Dahanayake 2019_b  doi:10.1111/lam.13229 | Clams | Korea | Retail | January to May 2018 | Vibrio | 31 | 0.59 | Disc diffusion test | P; TET; PM; AP; AM; M; Q; FQ; C; SXT; R; 3-4-5 GC; 1-2 GC; L |
| Hossain 2019  doi: 10.1089/mdr.2019.0131 | Mussels | Korea | Retail | January to December 2018 | Vibrio | 32 | 0.14 | Disc diffusion test | P; TET; PM; AP; AM; M; Q; FQ; C; SXT; 3-4-5 GC; 1-2 GC |
| Sacramento 2019  doi:10.1016/j.marpolbul.2019.04.014 | Mussels | Brazil | Environment | November 2016 | Enterococcus | 1 | 0.56 | MIC/Disc diffusion test | P; AP; AM; M; FQ; R; GP; O; ST |
| Dahanayake 2019_c  doi:10.1111/jam.14355 | Clams | Korea | Retail | January to May 2018 | Aeromonas | 36 | 0.39 | Disc diffusion test | P; TET; AP; AM; Q; FQ; C; SXT; 3-4-5 GC; 1-2 GC |
| Kurdial-Dulaimi 2019  doi:10.3390/antibiotics8020068 | Cockles, Clams | Malaysia/Qatar | NA | July 2013 to February 2014 | Vibrio | 60 | 0.45 | Disc diffusion test | P; TET; AM; M; 3-4-5 GC; 1-2 GC; L; GP; PP; AC |
| Lozano-Leon 2019_a  doi:10.2807/1560-7917.ES.2019.24.24.1900200 | Mussel | Spain | Aquaculture | 2012 to 2016 | Salmonella | 15 | 0.22 | MIC | P; AM; Q; FQ; C; SXT; 3-4-5 GC; 1-2 GC; βLI; FOS; N; GC |
| Ashrafudoulla 2019  doi:10.3389/fmicb.2019.00513 | Mussel | South Korea | Aquaculture | September to October 2016 | Vibrio | 8 | 0.78 | Disc diffusion test | P; TET; AP; AM; M FQ; L; GP |
| De Silva 2019_a  doi:10.1111/jam.14106 | Scallops | Korea | Retail | NA | Aeromonas | 32 | 0.68 | Disc diffusion test | P; TET; PM; AP; AM M; Q; FQ; C; SXT; R; 3-4-5 GC; 1-2 GC; L; GP |
| Li 2019  doi: 10.1111/jfs.12650 | Clams | China | Retail | 2012 | Vibrio | 65 | 0.13 | Disc diffusion test | P; TET; AP; AM; Q; FQ; C; SXT; 3-4-5 GC; 1-2 GC; βLI; CP |
| Ben Hassena 2019  doi:10.4315/0362-028X.JFP-18-607 | Clams | Tunisia | Environment | January 2011 to March 2012 | Salmonella | 7 | 0.00 | Disc diffusion test | Q; FQ; SXT; 3-4-5 GC; βLI |
| De Silva 2019_b  doi: 10.1111/jfs.12634 | Scallops | Korea | Retail | January to March 2018 | Vibrio | 30 | 0.49 | Disc diffusion test | P; TET; PM; AP; AM; M; Q; FQ; C; SXT; R; 3-4-5 GC; 1-2 GC; L; GP |
| Lee 2019  doi: /10.1016/j.aquaculture.2018.10.028 | Clams | Taiwan | NA | NA | Shewanella | 1 | 0.20 | MIC | AM; FQ; C; 3-4-5 GC; βLI |
| Ryu 2019  doi: 10.1007/s11356-019-05426-1 | Oysters, Mussels Short-necked clams, Ark shells | Korea | Aquaculture | February to November 2016 | Vibrio | 288 | 0.27 | Disc diffusion test | P; TET; AP; AM; Q; FQ; C; SXT; 3-4-5 GC; 1-2 GC; βLI; T; MO; |
| Agnoletti 2019  doi: 10.1016/j.ijfoodmicro.2019.03.003 | Mussels, Clams | Italy | Environment | December 2015 to August 2017 | Clostridium | 113 | 0.10 | E-test | M; FQ; R; L; GP; I |
| Miotto 2019  doi: 10.1002/mbo3.738 | Oysters | Brazil/USA | Environment | January to July of 2015/ October and November of 2015 | Escherichia | 141 | 0.37 | Disc diffusion test | P; TET; AP; AM; Q; FQ; SXT; 3-4-5 GC; 1-2 GC; βLI; N |
| Kang 2018  doi: 10.1016/j.marpolbul.2018.07.007 | Oysters | Korea | Aquaculture | May to October 2016 | Vibrio | 59 | 0.62 | Disc diffusion test | P; TET; AP; AM; M; Q; FQ; SXT; R; 3-4-5 GC; 1-2 GC; GP |
| Banerjee 2018  doi:10.1128/AAC.00799-18 | Oysters, Mussels, Clams | Canada | Aquaculture/Environment | May to October from 2006 to 2012 | Vibrio | 1021 | 0.07 | Disc diffusion test | P; TET; PM;L AP; AM; M; Q; FQ; SXT; 3-4-5 GC; 1-2 GC; S |
| Sellera 2018  doi:10.1016/j.jgar.2018.06.010 | Mussels | Brazil | Environment | 2016 | Escherichia | 1 | 0.27 | Disc diffusion test/E-test/MIC | TET; PM; AM; Q; FQ; C; SXT; 3-4-5 GC; 1-2 GC; βLI; MO |
| Lopatek 2018  doi:10.1128/AEM.00537-18 | Clams, Mussels, Oysters, Scallops, Razor shells, Cockles | Netherlands, Italy, Norway, France, Turkey, Denmark, Spain, Germany, Sri Lanka | Retail | 2009 to 2015 | Vibrio | 92 | 0.30 | MIC | P; TET; AP; AM; FQ |
| Vu 2018  doi:10.4315/0362-028X.JFP-18-029 | Blue mussels, Cockles, Razor shells, Venus clams | Bangladesh, Denmark, Ecuador, France, Germany, India, Ireland, Italy, Netherlands, Spain, Vietnam | Retail | December 2015 to August 2016 | Hafnia | 3 | 0.00 | Disc diffusion test | 3-4-5 GC; 1-2 GC |
| Silva 2018  doi:10.1016/j.marpolbul.2018.05.007 | Oysters, Mussels | Brazil | Environment/Retail | NA | Vibrio | 13 | 0.24 | Disc diffusion test/MIC | P; TET; AP; AM; Q; FQ; C; SXT; 3-4-5 GC; 1-2 GC; N; |
| Li 2018  doi:10.4315/0362-028X.JFP-17-357 | Mussels, Cockles | Germany | Retail | September 2015 to April 2016 | Yersinia | 2 | 0.20 | Disc diffusion test | P; TET; AP; AM; Q; FQ; 3-4-5 GC; 1-2 GC; T; MO; |
| Maravic 2018  doi:10.1007/s10661-018-6471-3 | Mussels | Croatia | Environment | October 2009 to November 2016 | Pseudomonas | 108 | 0.11 | Disc diffusion test | P; AM; FQ; C; SXT; 3-4-5 GC; βLI; MO |
| Al-Sarawi 2018  doi: 10.1016/j.marpolbul.2017.10.044 | Clams | Kuwait | Environment | July to August 2015, December 2015 to February 2016 | Escherichia | 247 | 0.22 | MIC | P; AM; FQ; C; SXT; 3-4-5 GC; 1-2 GC; βLI; N; MO |
| Dahanayake 2018  doi:10.1111/jfs.12490 | Oysters | Korea | Retail | October-November 2017 | Vibrio | 41 | 0.33 | Disc diffusion test | P; AM; Q; FQ; SXT; 1-2 GC; GP |
| Othman 2018  doi:10.1016/j.foodcont.2018.02.045 | Mussels, Cockles | Malaysia | Retail | January to May 2017 | Staphylococcus | 7 | 0.24 | Disc diffusion test | P; TET; AP; AM; M; FQ; SXT; ; 3-4-5 GC; 1-2 GC; O; βLI |
| Serratore 2017  doi:10.4081/ijfs.2017.6843 | Clams | Italy | Environment | NA | Vibrio | 5 | 0.10 | Disc diffusion test | P; TET; AP; AM; FQ; C; SXT; 3-4-5 GC; 1-2 GC; βLI |
| Yang 2017  doi:10.3389/fmicb.2017.02566 | Oysters | China | Retail | July 2015 to July 2017 | Vibrio | 34 | 0.24 | Disc diffusion test | P; TET; AP; AM; FQ; C; SXT; 3-4-5 GC; 1-2 GC |
| Olukemi Adesiji 2017  doi:10.7555/JBR.31.20160094 | Clams | India | Retail | NA | Salmonella | 28 | 0.46 | Disc diffusion test/MIC | P; TET; AP; AM; Q; FQ; C; SXT; βLI; N |
| Baron 2017  doi:10.3389/fmicb.2017.01637 | Cockles | France | Environment | June and October 2000 and September 2001 | Vibrio | 51 | 0.05 | Disc diffusion test | P; TET; AP; AM; M; Q; FQ; C; SXT; 3-4-5 GC; βLI; S |
| Lo 2017  doi:10.4315/0362-028X.JFP-16-336 | Oysters | Taiwan Island, USA, Chile, Canada, France, Japan, Korea | Retail | June to August 2010 / November 2010 to May 2011 | Salmonella | 91 | 0.05 | Disc diffusion test | P; TET; AP; AM; Q; SXT |
| Kang 2017  10.1016/j.marpolbul.2017.02.070 | Oysters | Korea | Aquaculture | June to October 2015 | Vibrio | 44 | 0.52 | Disc diffusion test | P; TET; AP; AM ;M; Q; FQ; SXT; R; 3-4-5 GC; 1-2 GC; GP |
| Rubiao 2017  doi:10.1590/1678-4324-2017160127 | Oysters | Brazil | Retail | NA | Enterococcus | 93 | 0.16 | Disc diffusion test | P; TET; AM; FQ; GP; O; N |
| Morejon 2017  doi:10.1089/fpd.2016.2202 | Mussels, Clams, Cockles | Spain | Retail | September 2013 to June 2015 | Arcobacter | 25 | 0.12 | Disc diffusion test/E-test | FQ |
| Odeyemi 2017  doi:10.1016/j.sjbs.2015.09.016 | Bivalves | Malaysia | Aquaculture | NA | Aeromonas | 10 | 0.53 | Disc diffusion test | P; TET; AP; AM; Q; AC; T |
| Kang 2016_a  doi:10.1007/s11356-016-7426-2 | Oysters | Korea | Aquaculture | July to October 2014 | Vibrio | 15 | 0.45 | Disc diffusion test | P; TET; AP; AM; M; Q; FQ; SXT; R; 3-4-5 GC; 1-2 GC; GP |
| Janecko 2016  doi:10.3201/eid2209.160305 | Clams | Vietnam | Retail | January to April 2015 | Enterobacter | 5 | 0.52 | MIC | P; TET; AP; AM; M; Q; FQ; SXT; 3-4-5 GC; 1-2 GC; βLI; S |
| Vignaroli 2016  doi: 10.1016/j.fm.2016.05.003 | Clams | Italy | Environment | April 2013 to July 2014 | Escherichia | 141 | 0.09 | Disc diffusion test | P; TET; AP; AM; Q; FQ; SXT; |
| Latif-Eugenin 2016  doi:10.1007/s00203-016-1189-5 | Mussels, Oysters | Spain | Environment | NA | Aeromonas | 1 | 0.00 | Disc diffusion test | P; TET; AM; FQ; C; SXT; 3-4-5 GC; 1-2 GC; |
| Mala 2016  doi:10.1016/j.meegid.2016.01.006 | Cockles | Thailand | Retail/Aquaculture | March 2010 to January 2012 | Vibrio | 74 | 0.10 | Disc diffusion test | P; TET; AP; AM; Q; FQ; SXT; 3-4-5 GC |
| Kang 2016_b  doi:10.1007/s11356-015-5650-9 | Oysters | Korea | Aquaculture | June to October 2014 | Vibrio | 71 | 0.54 | Disc diffusion test/MIC | P; TET; AP; AM; M; Q; FQ; SXT; R; 3-4-5 GC; 1-2 GC; GP |
| Kurdial-Dulaimi 2016  Asian Jr. of Microbiol. Biotech. Env. Sc. Vol. 18, No. (2) : 2016 : 25-34 | Cockles | Malaysia | Retail | July 2013 and February 2014 | Vibrio | 20 | 0.50 | Disc diffusion test | P; TET; AM; M; 3-4-5 GC; 1-2 GC; L; GP; PP; AC; |
| Lou 2016  doi:10.1016/j.foodcont.2015.04.039 | Oysters | China | Retail | 2009 to 2013 | Vibrio | 1 | 0.20 | Disc diffusion test | P; TET; AP; AM; M ;FQ; C; SXT; 3-4-5 GC; 1-2 GC; βLI |
| Yu 2016  doi:10.1016/j.foodcont.2015.08.005 | Clams, Oyster, Razor clams, Scallops | Shanghai | Retail | February 2014 and February 2015 | Vibrio | 96 | 0.23 | Disc diffusion test | P; TET; AP; AM; Q; FQ; C; SXT; 3-4-5 GC; 1-2 GC; βLI; MO |
| Bier 2015  doi:10.3389/fmicb.2015.01179 | Bivalves | Germany | Environment/Retail | 2004 to 2014 | Vibrio | 30 | 0.11 | Disc diffusion test/MIC | P; TET; PM; AP; AM; Q; FQ; C; SXT; 3-4-5 GC; βLI; T |
| Costa 2015  doi:10.1590/S0036-46652015000300002 | Oysters | Brazil | Retail | 2010 | Vibrio | 87 | 0.30 | Disc diffusion test | P; TET; AP; AM; M; Q; FQ; GP |
| Zhang 2015  doi:10.1016/j.ijfoodmicro.2015.04.019 | Razor shells | China | Retail | 2006-2011 | Salmonella | 72 | 0.22 | Disc diffusion test | P; TET; AP; AM; Q; FQ; SXT; ; 3-4-5 GC; βLI; S; T |
| Troiano 2015  doi:10.1016/j.ijfoodmicro.2015.05.002 | Mussels, Clams | Italy | Retail/Aquaculture/Environment | January 2012 to January 2014 | Clostridium | 36 | 0.24 | MIC agar dilution method; E-test | M; FQ; L; GP; I; MC; PT |
| Lopatek 2015  doi:10.4315/0362-028X.JFP-14-437 | Oysters, Clams, Mussels, Scallops | Poland, Netherlands, Norway, Italy, France | Retail | 2009 to 2012 | Vibrio | 64 | 0.33 | MIC | P; TET; AP; AM; FQ |
| Rees 2015  doi:10.1089/fpd.2014.1840 | Oysters, Mussels | Canada | Aquaculture/Environment | May to July 2012 | Escherichia | 22 | 0.09 | MIC | TET; AP; AM; Q; FQ; SXT; 1-2 GC; S |
| Yang 2015  doi: 10.1016/j.foodcont.2015.03.046 | Oysters | China | Retail | July 2011 to May 2014 | Salmonella | 6 | 0.22 | Disc diffusion test | P; TET; AP; AM; Q; FQ; SXT; 3-4-5 GC; 1-2 GC; βLI |
| Adesiji 2014  doi:10.1002/fsn3.119 | Clams | India | Retail | NA | Salmonella | 8 | 0.11 | Disc diffusion test | P; TET; AP; AM; Q; FQ; C; SXT; 3-4-5 GC; βLI; N |
| Mala 2014  doi:2014 Jan;45(1):103-12 | Cockles | Thailand | Retail/Aquaculture | NA | Vibrio | 40 | 0.03 | Disc diffusion test | P; TET; AP; AM; Q; FQ; SXT; 3-4-5 GC |
| Norman 2014  doi:10.1080/19440049.2014.888785 | Mussels, Clams, Oysters | USA | Retail | March, April, December 2012 | Clostridium | 1 | 0.20 | E-test | P; TET; AP; FQ; C; 1-2 GC; L; GP; βLI; I |
| Sahilah 2014  doi:10.1007/s11274-013-1494-y | Cockles | Malaysia | Environment | NA | Vibrio | 37 | 0.30 | Disc diffusion test | P; AP; AM; FQ; 3-4-5 GC; 1-2 GC; GP; PP |
| Collado 2014  doi:10.1016/j.foodcont.2014.06.013 | Mussels, Clams, Oysters, Scallops, Razor shells | Chile | Retail | July 2010 to March 2016 | Arcobacter | 99 | 0.23 | MIC/Agar dilution method | P; AM; M; Q; FQ |
| Soultos 2014  doi:10.3906/vet-1212-36 | Mussels | Greece | Aquaculture/Environment | NA | Listeria | 1 | 0.44 | Disc diffusion test | P; TET; AP; AM; M; Q; SXT; 1-2 GC; GP |
| Kang 2013  doi:10.1016/j.marpolbul.2013.09.025 | Mussels, Clams, Oysters | Korea | Environment | May 2012 to December 2012 | Shewanella | 23 | 0.25 | Disc diffusion test | P; TET; AP; AM; M; Q; FQ; SXT; R; 3-4-5 GC; 1-2 GC; GP |
| Batista 2013  doi:10.1089/fpd.2013.1576 | Clams | Brazil | Environment/Retail | April to June 2009/ Jan to March 2012 | Staphylococcus | 79 | 0.24 | Disc diffusion test/MIC | P; TET; AP; AM; M; FQ; R; 1-2 GC; L |
| Maravic 2013_a  doi:10.1016/j.ijfoodmicro.2013.07.010 | Mussels | Croatia | Environment | June to July 2009-2010 | Aeromonas | 147 | 0.24 | Disc diffusion test/E-test/MIC | P; C; 3-4-5 GC; βLI; CP; MO |
| Collin 2013  doi:10.1111/j.1574-6941.2012.01471.x | Clams | Mozambique | Environment | November - February - March -May | Vibrio | 109 | 0.25 | Disc diffusion test | P; TET; AP; AM; Q; FQ; SXT; 1-2 GC |
| Sayd 2012  doi:10.4014/kjmb.1205.05002 | Mussels | India | Environment | NA | Vibrio | 15 | 0.55 | Disc diffusion test | P; TET; AP; AM; Q; FQ; C; SXT; R; 1-2 GC; N; CP; S; T |
| Rojas 2011  doi:10.1590/S0036-46652011000400005 | Oysters, Mussels | Brazil | Environment/Retail | February 1989 to January 1990 | Vibrio | 19 | 0.18 | Disc diffusion test | P; TET; AP; AM; Q; FQ; C; SXT; 3-4-5 GC |
| Brillhart 2011  doi:10.4315/0362-028X.JFP-10-443 | Oysters | Arizona (USA) | Retail | October, December 2007, February, April, June to September 2008 | Salmonella | 28 | 0.26 | Disc diffusion test | P; TET; AP; AM; Q; FQ; SXT; 1-2 GC; βLI |
| Kim 2011  doi:10.1111/j.1745-4565.2011.00329.x | Clams, Mussels, Razor shells, Oysters | Korea | Retail | May to December 2009 | Vibrio | 13 | 0.47 | Disc diffusion test | P; TET; AP; AM; FQ; C; SXT; 3-4-5 GC; 1-2 GC; βLI |
| Valenzuela 2010  doi:10.1016/j.fm.2010.05.033 | Clams, Mussels, Cockles, Razor shells, Oysters | Spain | Retail | 6 months | Enterococcus | 7 | 0.10 | MIC | P; TET; AP; AM; M; FQ; R; GP; ST; N |
| Soonthornchaikul 2009  doi:10.1089/fpd.2008.0236 | Cockles, Mussels, Oysters | Thailand | Retail | 40 days | Campylobacter | 335 | 0.36 | Epsilometer test | M; Q; FQ; |
| Doublet 2009  doi:10.1128/AAC.01581-08 | Cockles | France | Retail | August to September 2005 | Salmonella | 4 | 0.27 | Disk diffusion test/MIC/E-test/Double Disk synergy test | P; TET; AP; AM; Q; FQ; C; 3-4-5 GC; 1-2 GC; βLI; CP; S; T; MO; AY |
| Liu 2009  doi:10.1248/jhs.55.783 | Mussels, Oysters, Clams | China | Environment | NA | Vibrio | 13 | 0.52 | MIC | P; AP; AM; FQ; SXT; 3-4-5 GC; 1-2 GC; N; S; |
| Ponce 2008  doi:10.1016/j.fm.2007.09.001 | Mussels | China | Retail | 2005 | Salmonella | 1 | 0.00 | Disc diffusion test | P; TET; AP; AM; S |
| Watkinson 2007  doi:10.1128/AEM.00763-07 | Oysters | Australia | Environment | NA | Escherichia | 50 | 0.02 | Disc diffusion test | P; TET; AM; Q; 1-2 GC; S |
| Han 2007  doi:10.1128/AEM.01116-07 | Oysters | USA | Environment/Retail | 2005-2006 | Vibrio | 319 | 0.06 | MIC | P; TET; AP; AM; FQ; C; 3-4-5 GC |
| Evangelista-Barreto 2006  doi:10.1590/s0036-46652006000300003 | Oysters | Brazil | Environment | April to October 2002 | Aeromonas | 59 | 0.09 | Disc diffusion test | TET; AP; Q; FQ; SXT; 3-4-5 GC; 1-2 GC; N |
| Brands 2005  doi:10.1089/fpd.2005.2.111 | Oysters | USA | Retail | NA | Salmonella | 78 | 0.38 | E-test | P; TET; AM; FQ; SXT |
| Ottaviani 2005  doi:10.1016/j.fm.2005.08.001 | Mussels | Italy | Environment | December 2002 to 2004 | Aeromonas | 24 | 0.47 | Disc diffusion test | P; TET; PM; AP; AM; Q; FQ; C; SXT; R; ; 3-4-5 GC; 1-2 GC; L; AC; N; CP; S; T |
|  | Mussels | Italy | Environment | December 2002 to 2004 | HG2 | 8 | 0.43 | Disc diffusion test | P; TET; PM; AP; AM; Q; FQ; C; SXT; R; ; 3-4-5 GC; 1-2 GC; L; AC; N; CP; S; T |
| Guardabassi 2004  doi:10.1128/AEM.70.2.984–990.2004 | Mussels | Denmark | Environment | October 2001 to August 2002 | Enterococcus | 1 | 0.50 | Disc diffusion test | P; TET; AP; M; FQ; GP; |
| Martinez-Urtaza 2004  doi:10.1128/AEM.70.7.4030-4034.2004 | Mussels,Oysters,  Clams | Spain | Aquaculture | 1998-2002 | Salmonella | 23 | 0.09 | Disc diffusion test | P; TET; PM; AP; AM; Q; SXT; 3-4-5 GC; βLI; N; S |
| Ripabelli 2003  doi:10.1078/072320203322337407 | Mussels | Italy | Retail | NA | Vibrio | 23 | 0.53 | Disc diffusion test | P; AM; M;FQ; SXT; 3-4-5 GC; CP |
| Wilson 2002  doi:10.1016/s0168-1605(02)00063-6 | Oysters, Mussels, Cockles | Northern Ireland | Environment | June to October 1998, September 1999 to January 2000 | Enterococcus | 22 | 0.27 | E-test | GP |
| Radu 1998  doi:10.1111/j.1574-6968.1998.tb13138.x | Cockles | Malaysia | Retail | NA | Vibrio | 25 | 0.62 | Disc diffusion test | P; TET; AP; AM; M; Q; CP; |
| Sathiyamurthy 1997  doi:10.1089/mdr.1997.3.267 | Mussels, Clams, Oysters | India | Environment | NA | Vibrio | 71 | 0.13 | Disc diffusion test | P; TET; PM;AP; AM; M; 1-2 GC; S; T |
| Weber 1994  doi:10.1017/s0950268800057368 | Bivalves | Ecuador | Retail | 1991 | Vibrio | 1 | 0.00 | MIC | P; TET; AP; AM; M; Q; FQ; SXT; N; S |
| Jabar Al Mossawi 1983  doi: 10.1007/BF00284629 | Clams | Kuwait | Environment | NA | Escherichia | 12 | 0.42 | Disc diffusion test | P; TET; AP; AM; S; T |

**ABBREVIATION:** P = Penicillins; TET = Tetracyclines; PM = Polymyxins; AP = Amphenicols; AM = Aminoglycosides; M = Macrolides; Q = quinolones; FQ = fluoroquinolones; C = Carbapenemes; SXT = Trimethoprim - sulfonamide combinations; R = Rifamycins; 1-2 GC = First/Second generation of cephalosporins; 3-4-5 GC = Third/Fourth/Fifth generation of cephalosporins; L = Lincosamides; GP = Glycopeptides; O = Oxazolidinones; ST = Streptogramins; PP = Polypeptides; AC = Aminocoumarin; βLI = Beta lactam - beta lactamase inhibitor; FOS = Phosphonics; N = Nitrofurans; GC = Glycylcyclines; CP = Carboxypenicillins; S = Sulphonamides; MO = Monobactams; T = Trimethoprim; I = Imidazoles; MC= Macrocyclic; PT = Peptides; AY = Aminocyclitols.

MAR = multiple antibiotic resistance index
